# The RNA chaperone StpA enables fast RNA refolding by destabilization of mutually exclusive base pairs within competing secondary structure elements

**DOI:** 10.1093/nar/gkab876

**Published:** 2021-10-06

**Authors:** Katharina F Hohmann, Anja Blümler, Alexander Heckel, Boris Fürtig

**Affiliations:** Institute for Organic Chemistry and Chemical Biology, Center for Biomolecular Magnetic Resonance BMRZ, Goethe University Frankfurt am Main, Max-von-Laue-Strasse 7, 60438 Frankfurt/Main, Germany; Institute for Organic Chemistry and Chemical Biology, Goethe University Frankfurt am Main, Max-von-Laue-Strasse 7, 60438 Frankfurt/Main, Germany; Institute for Organic Chemistry and Chemical Biology, Goethe University Frankfurt am Main, Max-von-Laue-Strasse 7, 60438 Frankfurt/Main, Germany; Institute for Organic Chemistry and Chemical Biology, Center for Biomolecular Magnetic Resonance BMRZ, Goethe University Frankfurt am Main, Max-von-Laue-Strasse 7, 60438 Frankfurt/Main, Germany

## Abstract

In bacteria RNA gene regulatory elements refold dependent on environmental clues between two or more long-lived conformational states each associated with a distinct regulatory state. The refolding kinetics are strongly temperature-dependent and especially at lower temperatures they reach timescales that are biologically not accessible. To overcome this problem, RNA chaperones have evolved. However, the precise molecular mechanism of how these proteins accelerate RNA refolding reactions remains enigmatic. Here we show how the RNA chaperone StpA of *Escherichia coli* leads to an acceleration of a bistable RNA’s refolding kinetics through the selective destabilization of key base pairing interactions. We find in laser assisted real-time NMR experiments on photocaged bistable RNAs that the RNA chaperone leads to a two-fold increase in refolding rates at low temperatures due to reduced stability of ground state conformations. Further, we can show that upon interaction with StpA, base pairing interactions in the bistable RNA are modulated to favor refolding through the dominant pseudoknotted transition pathway. Our results shed light on the molecular mechanism of the interaction between RNA chaperones and bistable RNAs and are the first step into a functional classification of chaperones dependent on their biophysical mode of operation.

## INTRODUCTION

Modulation of gene expression in bacteria can be provided by RNA regulators. These RNAs functionally exploit their inherent structural plasticity that is founded in their natural ability to adopt more than a single stable conformation. Thereby they constitute a bi- or even multi-stable system. If the structural interchangeability within such a system takes place as an answer to changes of environmental clues, it can be exploited as a trigger for functional cellular processes such as transcription or translation. Within the regulation of gene expression, the conformational states of a bistable system are typically linked to two functional states (ON and OFF) (1,2). Prime examples for such biological bistable RNAs are RNA thermometers and riboswitches found in the 5′- or 3′-untranslated regions of mRNAs (2–6). For both types of RNA regulators, the basic functional principle can be broken down to an equilibrium of conformations that is characterized by mutually exclusive base pairs in the two states. In general, the involved states have similar thermodynamic stability rendering the difference in free energy close to zero, and thereby facilitating a change in the population ratio by environmental clues.

**Figure 1.**
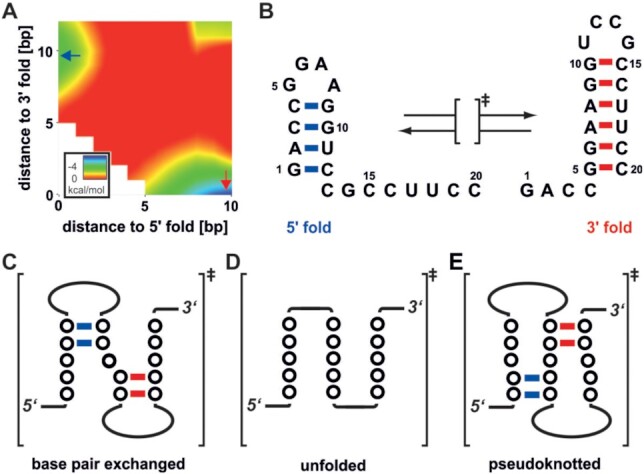
Bistable RNA refolding. (**A**) Mean free energy profile of the bistable RNA system as calculated by RNA2Dfold (7), estimating the energy barrier between the two conformational ground states. (**B**) Sequence and folding states (5′-fold and 3′-fold) of the bistable 20 nt RNA. From a biophysical perspective refolding between these two states can in principle occur through three different transition states that are described as (**C**) two base paired exchanged stems, (**D**) unfolded chain of nucleotides or (**E**) a pseudoknotted conformation.

Bistable RNAs are designed mimicry of such biological RNAs in conformational equilibrium. They are studied with a variety of different NMR spectroscopic methods in order to understand the structural basis of conformational switching and refolding in RNAs (8–10). The dominant possible refolding mechanisms for bistable RNAs are the refolding after complete unfolding, the folding through base pair exchange pathways and the pseudoknot-assisted pathway (Figure 1C–E) (11,12). However, the refolding of an RNA from a stable conformational state into another is not only a highly temperature-dependent process but most often occurs on nonbiological long time scales (seconds to minutes) *in vitro* (13).

While folding of secondary structural elements and the subsequent formation of tertiary interactions is fast when starting from unfolded states (10–100 μs and 1–100 ms, for 2° and 3° interactions, respectively), refolding occurs at timescales of 1–1000 s (14,15). However, the refolding of native bistable regulatory RNA elements has to proceed on a time scale that is accommodated by the timescale of the bacterial live cycle. If for example the doubling time of the bacteria is of 20 minutes (as example for *E. coli*) then all regulatory processes have to be faster in order to have a relevant functional consequence (16).

In order to accelerate RNA refolding kinetics *in vivo* bacteria exhibit a class of auxiliary proteins: RNA chaperones (15,17). This sequentially and structurally diverse set of proteins unfolds RNAs non-specifically or hinders the formation of misfolded RNA structures (15,18). The function of RNA chaperones is based on their transient binding to RNAs and the induced disruption of secondary and tertiary structures. By unwinding and unfolding of structural elements the formation of alternative base pairing interactions and consequently refolding is accelerated (19). Chaperones do not need an external energy source such as ATP, and can therefore be distinguished from helicases that are also assisting RNA folding processes (20). The most prominent representatives of RNA chaperones are cold shock proteins like CspA (21) and CspE (22), the Sm family protein Hfq (23), FinO (24), the ribosomal proteins S1 (25) and S12 (26) as well as H-NS and StpA (27,28). Some of these chaperones are shown to be essential for the refolding and function of RNAs *in vivo* (29), while others can be supplemented and do not show a severe phenotype after deletion (30). However, the mechanism and the underlying driving force remain mostly unclear. Most RNA chaperones act non-sequence specific, so that they are able to interact with various RNAs (26,31). Further, many of the chaperones interact with the RNA’s backbone through transient electrostatic interactions. However, other molecular features are important for the enhancement of RNA folding reactions beyond positive charges (32). The transient nature of the interactions results in a rapid release of the RNA, and in turn to a moderate affinity to folded RNAs (33). This depicts also the main difference to specific RNA binding proteins that stabilize a certain RNA fold by remaining bound (34). Many chaperones have in common a high density of disordered or dynamic regions that are important for their activity. This led to the proposition of the entropy transfer model that defines the disorder-order transition between chaperone and the RNA as the driving force (35). During the interaction the chaperone gets more rigid, whereas the RNA becomes more flexible, followed by refolding of the RNA. In this regard an increase in the internal RNA dynamics could be observed upon interaction with the cold shock protein CspA. It increased the hairpin refolding rates through destabilizing of base pairs that are close to the loop or at a helix junction (21).

Here, we are addressing the chaperone induced changes on the refolding mechanism of a bistable RNA with regard to the thermodynamics and kinetics of the system. We are using the nucleoid-associated protein StpA from *E. coli* (36) as a model chaperone. The N-terminal domain of StpA exerts protein-protein interactions and the C-terminal domain (CTD) exclusively harbours the chaperone activity, even in isolation (28). As molecular model for the RNA, a well characterized 20 nt bistable RNA sequence was used, that adopts a temperature-dependent equilibrium between two interconverting hairpin structures (5′-fold, 3′-fold, Figure 1B) (8,12) The two different structures exhibit similar thermodynamic stabilities but are separated through a high energy barrier (Figure 1A) (7). The RNA was originally designed to highlight the fact that RNA sequences are inherently able to adopt multiple stable conformations and that this property can be exploited as molecular switches (37). The low energy difference between the two ground states was first experimentally validated by completely shifting the conformational equilibrium through the incorporation of few methyl groups within bases that are involved in mutually exclusive base-interactions (38). Such bistable RNAs are well suited models for biologically relevant RNAs, as refolding rates determined for bistable sequences can be used to describe the refolding behaviour of riboswitches (8,39). The switching kinetics of the I-A type 2′dG-sensing riboswitch from *Mesoplasma florum* could be modelled based on the refolding rates measured on bistable sequences, including the sequence investigated here. The modelled behaviour agrees with the experimentally derived kinetics of the riboswitch system and validates that refolding kinetics of bistable sequences indeed represent good models for biological RNA refolding events.

By NMR spectroscopy, we probe the influence of the RNA chaperone onto the equilibrium forming conformational states and the refolding mechanism. The binding of StpA to the RNA was investigated by isothermal titration calorimetry in order to establish the full thermodynamic cycle of the interaction. Further, the kinetics of their interconversion is studied by real-time NMR spectroscopy of photocaged RNAs. The results show the ability of StpA-CTD to accelerate RNA refolding kinetics by selective destabilization of base pair interactions.

## MATERIALS AND METHODS

### Synthesis of photocaged RNA and NMR sample preparation

The synthesis of the (*S*)*-*NPE protected guanosine phosphoramidite was performed according to literature procedures (8) (NMR Spectra and synthesis steps see SI, Scheme S1, Supplementary Figures S1–S6, Supplementary Tables S1 and S2). The oligonucleotide **RNA** was synthesized on an *ABI392 DNA/RNA synthesizer* from *Applied Biosystems* or on an *Oligopilot OP10* plus from *GE Healthcare*. 0.3 M BTT from *emp BIOTECH* was used as activator. The oligonucleotide was synthesized and deprotected under *UltraMILD* conditions (Pac-rA-CE, *i*PrPac-rG-CE, Ac-rC-CE, U-CE and Ac-C-CE RNA SynBase™ 1000 Å CPG purchased from *Glen Research* and *Linktech*) in 1 μmol or 16 μmol scales. Pac_2_O was used as capping reagent. The coupling time was 12 min. **RNA** was synthesized in DMTr-On mode. Deprotection was performed according to a protocol from *Glen Research* (40). The cyanoethyl groups were removed by flushing the solid phase column with 20% diethylamine in MeCN for 10 min, followed by washing the CPG with MeCN. For the resin cleavage and further deprotection, the solid phase material was treated with 400 μl ammonium hydroxide and EtOH (3:1, v/v) for 4 h at room temperature. After evaporation of the solvent at 4°C, the residue was dissolved in 115 μl DMSO and the 2′-TBDMS groups were cleaved by incubation with 60 μl Et_3_N and 75 μl Et_3_N·3HF at 60°C for 2.5 h. The fully deprotected RNA was precipitated by adding 25 μl 3 M NaOAc and 1 ml prechilled *n*-butanol overnight at -20°C and pelleted by centrifugation at 4°C for 30 min. The precipitation steps were repeated to completely remove fluoride salts. The crude product was purified *via* RP-HPLC using an *Agilent 1200 series* instrument with an *XBridge Peptide BEH C18 OBD Prep Column* (300Å, 5 μm, 10 × 250 mm, 4.0 ml min^–1^, 60°C) from *Waters* (RP-HPLC conditions see SI). After evaporation of the solvent at 4°C the DMTr-group was cleaved by incubation of the RNA in 300 μl 80% acetic acid at room temperature for 20 min. The solvent was evaporated under vacuum and the resulting RNA was again purified by RP-HPLC using the same conditions as mentioned before. The solvent was removed under vacuum. Finally, all samples were coevaporated with ultrapure water several times and lyophilized (Sequence and ESI-MS results see SI). Buffer exchange to NMR buffer (50 mM BisTris, 25 mM NaCl, pH 6.4) was done with 2 ml 2 kDa centrifugal concentrator (Vivaspin from Sartorius) up to a factor of at least 1000. The NMR sample was *in situ* folded at 95°C for 5 min followed by cooling on ice.

### Unmodified RNA NMR sample Preparation

The unmodified bistable 20 nt RNA and the supplementary RNAs (3′-HP, A2-DMA, 5′-HP, G6-m1G, 5′-ssOV) were solid phase synthesized and purchased by Dharmacon (horizon inspired call solutions). 2′-ACE Protection group was removed as described in the provided protocol by Dharmacon. After deprotection the RNA was purified via rp-HPLC (reversed-phase high-performance liquid chromatography) with a Kromasil RP18 100A 5 μm 10 × 250 mm column (binding buffer: 2 mM Tetrabutylammoniumbisulfat, 50 mM KH_2_PO_4_/K_2_HPO_4_ pH 5.9, elution buffer: binding buffer + 60% (v/v) Acetonitril). The HPLC fractions were lyophilized and dissolved in H_2_O. Analytical denaturing 15%-PAA (polyacrylamide) gel verified corresponding fractions that were desalted with 2 ml 2 kDa centrifugal concentrator (Vivaspin, Sartorius) at 4°C and 6000 g. After desalting LiClO_4_ (2% LiClO_4_ in acetone) precipitation followed. Buffer exchange to NMR buffer (50 mM BisTris, 25 mM NaCl, pH 6.4) was done with 2 ml 2 kDa centrifugal concentrator (Vivaspin from Sartorius) up to a factor of at least 1000. The NMR sample was *in situ* folded at 95°C for 5 min followed by cooling on ice. For sequences and sample conditions of all RNAs used see SI Supplementary Table S4 and Table S6)

### Cloning, expression and purification of StpA-CTD

The StpA-CTD sequence was cloned into a pE-SUMOstar Vector (LifeSensors #1106). The utilized plasmid carried the gene for ampicillin resistance. This vector provides a HIS_6_ tag at the N-terminus of the SUMOstar protein sequence behind which the StpA-CTD sequence is inserted. This allows purification by IMAC. The cloning was performed as described in the SUMOstar^®^ Gene Fusion Technology product manual. The pE-SUMOstar vector was digested with BsaI restriction endonuclease. The gene of interest was amplified by PCR with primers designed for the cloning strategy followed by digestion with BsaI restriction endonuclease (protein and primer sequences see SI Supplementary Table S3). Ligation of the prepared insert into the digested vector was done as described in the *S*UMOstar^®^*Gene Fusion Technology* product manual.

Plasmid transformation and amplification were performed using T7 Express High Efficiency *E. coli* cells from NEB (Ipswich, MA, USA) following the instructions of the high efficiency transformation protocol of the manufacturer. Purification of the plasmid was done with QIAGEN plasmid Kits (Cat. No. 12145). The plasmids containing the protein sequences were transformed into T7-Express *E. coli* cells for subsequent expression as SUMO-fusion protein. Cells were grown in TB (Terrific Broth) medium supplemented with 100 μg/m. ampicillin at 37°C. Expression was induced when OD_600_ reached a value between 2 and 2.5 with 0.1 mM IPTG (Isopropyl-β-d-thiogalactopyranosid), the optimal concentration evaluated with test expressions. Expression took place at 24°C, 120 rpm for 16 h. After harvest the pellet was resuspended to homogeneity in 50 ml buffer A (50 mM BisTris, 450 mM NaCl, 5 mM Imidazol, pH 7.0) supplemented with one protease-inhibitor tablet (cOmplete™, Roche, Germany) per liter. The cells were mechanically lysed using Microfluidics M-110P. Purification by IMAC HisTrap HP column (GE Healthcare, USA), connected to a FPLC (Äktapurifier™, GE Healthcare, USA) was done. Bound protein was washed with buffer A. To remove nucleic acid impurities, the protein was further washed with a high salt buffer (50 mM BisTris, 2 M LiCl, 5 mM imidazole, pH 7). With a linear gradient of imidazole (buffer b: 50 mM BisTris, 450 mM NaCl, 500 mM imidazole, pH 7) the fusion proteins of interest were eluted. Cleavage of the Tag with SUMO protease took place during dialysis against imidazole in buffer A and removage with reverse Ni-NTA affinity chromatography After reverse Ni-NTA 0.015% (w/v) of polyethyleneimine (PEI) was added to precipitate bound nucleic acids Due to impurities additional size-exclusion chromatography (SEC, 320 ml HiLoad 26/600 Superdex 75 pg gel filtration column (GE Healthcare. USA) with an NMR running buffer (50 mM BisTris, 25 mM NaCl, pH 6.4). Further purification followed by Heparin column (5 ml HiTrap® Heparin High Performance, GE Healthcare), to get rid of RNase activity. Therefore, binding buffer C containing the NMR buffer and 5% of glycerol was used (50 mM BisTris, 25 mM NaCl, pH 6.4, 5% glycerol) was used. Buffer exchange to NMR buffer without glycerol was achieved and purity was confirmed by SDS-PAGE (silver stained) and mass spectroscopy (MALDI Spectra of purified StpA-CTD see SI Supplementary Figure S7, NMR spectroscopic analysis see SI Supplementary Figure S8 and S9).

### NMR spectroscopy

NMR experiments were performed on Bruker NMR spectrometers with different probe heads listed in Supplementary Table S5. NMR experiments were performed with standard Bruker pulse sequences and spectra were recorded and analyzed with *TopSpin 3.5pl5-7*. All samples containing 10% D_2_O and the same buffer: 50 mM BisTris, 25 mM NaCl, pH 6.4. 1D ^1^H imino proton spectra were recorded using a jump return echo pulse sequence. Thermal equilibration for all samples at each temperature was done for at least 20 minutes before the experiments were recorded. NMR Titrations were performed at 25°C, StpA-CTD was added stepwise in molar ratios of [RNA]:[StpA-CTD] = 0, 1, 2, 3, 4, 5.

#### Real-time NMR

Real-time NMR experiments were conducted as described in Fürtig *et. al*. (8), however with the following modifications: a laser with a wavelength of 355 nm (Paladin Advanced 355-8000) was used for the photo reaction. Pseudo-2D ^1^H spectra were recorded with jump return echo for water suppression. The RNA samples were irradiated 1 s with a laser power of 4 W. The normalized kinetic data were fitted according to the integrated rate-laws for a reversible unimolecular reaction. The fit formula used are the following: 5′-fold 3′-fold $normalized\ signal\ {( {3^{\prime}} )_t} = \frac{K}{{K + 1}}\ *( {1 - K*{e^{ - {k_{5^{\prime} - 3^{\prime}\ }}t*( {1 + \frac{1}{K}} )}}} )$. With *K* for the equilibrium constant at the corresponding temperature for the unmodified RNA (see SI Supplementary Table S8). The k_3′-5′_ is calculated by $\frac{{{{{\bf k}}_{5^{\prime} - 3^{\prime}}}\ }}{K}$. The activation enthalpy is calculated with k_5′-3′_ and k_3′-5′_ by linearization and Arrhenius equation $\ln ( {{k_{5^{\prime} - 3^{\prime}\ }}} ) = \ - \frac{{\Delta {H^\ddagger }}}{{RT}} + \ln ( A )$ and $\ln ( {{k_{3^{\prime} - 5^{\prime}\ }}} ) = \ - \frac{{\Delta {H^\ddagger }}}{{RT}} + \ln ( A )$.

Integrity of the samples after kinetic experiments is verified mass-spectrometry and denaturing PAGE (see SI Supplementary Figure S15).

#### Measurement of base pair stabilities by NMR

Pseudo-2D water exchange NMR experiments were conducted and evaluated as described in Rinnenthal *et al.* (6). Therefore, the imino peak integrals were plotted against the inversion recovery delay τ_m_ and fitted resulting the exchange rate *k*_ex_. The enthalpy and entropy differences of the closed versus the open state of the nucleobases Δ*H*_diss_, Δ*S*_diss_ and Δ*G*_diss,_ were calculated from the exchange rates at different temperatures.

### Isothermal titration calometry (ITC)

All RNA constructs (20mer RNA, G6m1G, A2-DMA, 5′-HP, 3′-HP, 5′-SS-OV) and the RNA-chaperone StpA-CTD were prepared in the same buffer stock (as NMR buffer: 50 mM BisTris, 25 mM NaCl, pH 6.4). The RNA samples were in situ folded at 95°C for 5 min followed by cooling on ice. ITC experiments were carried out at 25°C and 5°C using a MicroCal iTC_200_ (Malvern Instruments). StpA with a concentration of 400/ 800/ 920 μM was injected into the cell containing 40 μM RNA (cell volume 201.9 μl). The initial waiting time was 120 s, followed by a first injection of 0.2 μl. Afterwards 19 injections of 2 μl within 4 s, with a delay of 180 s after each injection, followed. In each experiment, a reference power of 11 μcal s^–1^ and stirring speed of 750 rpm were set with a high feedback mode. For each construct, three titrations were measured and the presented corresponding *K*_D_ values and thermodynamic data represent the average with standard deviation, except the 5′-SS-OV that was measured once at 5°C and 25°C. Analysis of the thermograms was done with the software Origin7.0 (originLab) and were fitted to the following model (41): $Q\ = \frac{{n{M_t}\Delta H{V_0}}}{2}\ [ {1 + \frac{{{X_t}}}{{n{M_t}}} + \frac{1}{{nK{M_t}}}\, - \,\sqrt {{{( {1 + \frac{{{X_t}}}{{n{M_t}}} + \frac{1}{{nK{M_t}}}} )}^2}\, - \,\frac{{4{X_t}}}{{n{M_t}}}} } ]$ including the correction for the dilution in each titration step with

$$\begin{equation*}\Delta Q\ \left( i \right) = \ Q\left( i \right) + \frac{{d{V_i}}}{{{V_0}}}\left[ {\frac{{Q\left( i \right) + Q\left( {i - 1} \right)}}{1}} \right] - Q\left( {i - 1} \right)\end{equation*}$$

With *K* Binding constant, *n* Hill coefficient, *V*_0_ active cell volume, *M*_t_ bulk concentration of macromolecule in *V*_0_, *X*_t_ bulk concentration of ligand, ***Q*** total heat content of the solution contained in *V*_0_, Δ*H* molar heat of ligand binding, Δ*Q*(*i*) heat released from the *i*th injection, *Q*(*i*) heat content at the end of the *i*th injection, Δ*V*_*i*_ injection volume. As reference, the thermogram of the titration of StpA (921 μM) into buffer is given in SI Supplementary Figure S13.

## RESULTS

### Thermodynamics

**Figure 2.**
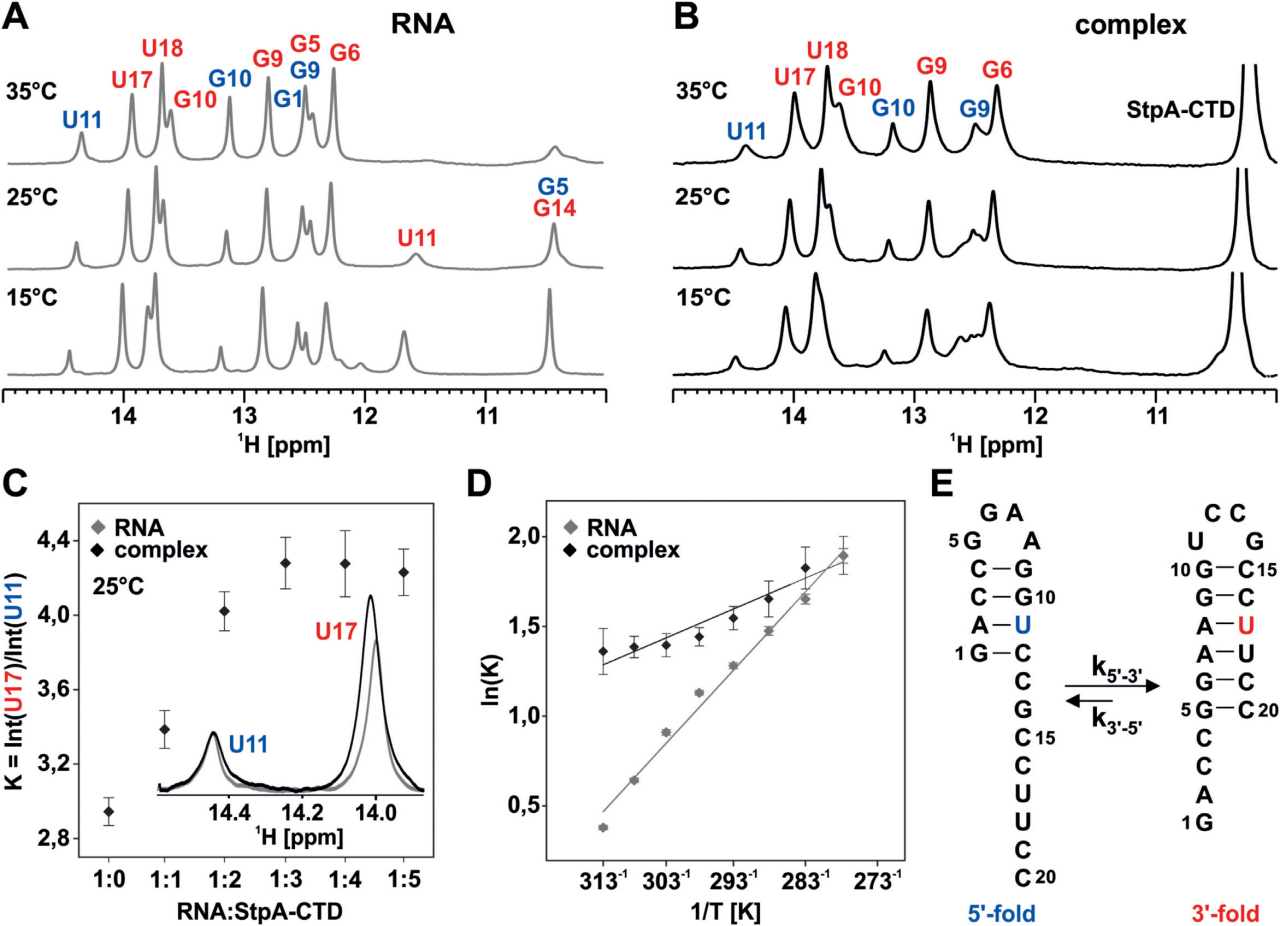
(A, B) Imino proton region of ^1^H NMR spectra of 20 nt RNA sequence at 15°C, 25°C and 35°C with color-coded resonance assignment (blue and red annotations indicate signals stemming from 5′- and 3′-fold, respectively). (**A**) Spectra of RNA alone (*c*_RNA_= 477 μM), (**B**) spectra of RNA (*c*_RNA_= 74 μM) in complex with 5 equivalents of the RNA chaperone StpA-CTD. (**C**) Change of equilibrium constant at 25°C upon titration with StpA-CTD; insert shows imino signals of U11 and U17 for the RNA alone (gray) and the 1:5 RNA:StpA-CTD complex (black). (**D**) Van’t Hoff Plot/Fit of ln(**K**) versus reciprocal temperature according to ln(*K*) = −Δ*H*°/*RT* + Δ*S*°/*R*, gray RNA alone; black RNA chaperone at complex ratio of 1:5. The error bars represent the SNR of each NMR experiment. Results of the fit: Δ*H*°^(RNA)^ = −30.1 ± 1.4 kJ/mol, Δ*S*°^(RNA)^ = −92 ± 5 J/mol, Δ*H*°^(RNA+3eq. StpA-CTD)^ = −11.87 ± 0.16 kJ/mol, Δ*S*°(RNA) = −27 ± 5 J/mol. (**E**) Secondary structure models of the two RNA conformations for 20 nt bistable RNA, color-coded 5′-fold (U11) and 3′-fold (U17). The error bars represent the signal-to-noise ratio (SNR) of each NMR experiment.

In order to determine the influence of the RNA Chaperone StpA-CTD on the thermodynamics of the bistable RNA, we investigated the conformational equilibrium of the RNA by NMR both in absence and presence of different amounts of the protein. The bistable RNA alone adopts independent on the ionic strength conditions two conformational states (8,38,42,43). A double set of signals in NMR spectra report on two distinct hairpin conformations that interconvert on a timescale longer than the acquisition time of the NMR experiment (Figure 2A, SI Supplementary Figure S11). The 5′-fold consists of a GGAA-tetraloop hairpin structure with four base pairs at the 5′-end and an eight nucleotides long single strand overhang. In contrast, the 3′-fold is build-up by a six base pair helix capped with an UCCG-tetraloop followed by four single stranded nucleotides (Figure 2E). The difference in free energy at 25°C is predicted to be 2.9 kJ/mol, which explains the coexistence of both structures (13). Calculated from the integration of isolated imino proton NMR signals of U11(3′)/U17(5′) the population ratio of 3′-fold/ 5′-fold was determined to be *K*^3^′^/5^′_10°C_ = 5.22 ± 0.09 to *K*^3^′^/5^′_40°C_ = 1.46 ± 0.04. This reports on a small energy separation of Δ*G*^3^′^/5^′_25°C_ = −2.60 ± 0.13 kJ/mol, which is in good agreement with the prediction. However, the population ratio is strongly temperature-dependent with a steep gradient of Δ*G*/d*T*^3^′^/5^′ = 92 ± 5 J/mol K. Also, upon addition of the RNA chaperone StpA-CTD two sets of signals are observable. Comparison with the signals of the RNA alone shows that there is no significant change of the individual chemical shifts and therefore the pattern of signals remains virtually identical. However, for all signals an increase in linewidth is observable and some are even broadened beyond detectability (Figure 2B). These are the signals from the loop and the end of the helix stem, G1/G5(5′-fold) and U11/G14(3′-fold), they are vanishing upon interaction with StpA-CTD. These findings regarding the imino proton signals are a first indication that StpA is not changing the dominant underlying refolding mechanism of the bistable RNA. If StpA forced the RNA into a dominant unfolding pathway, all resonances would be broadened beyond detectability. If the base pair exchange mechanism became dominant, a different set of signals would arise in conjunction with a differential modulation of signal intensities. However, the ratio of the two conformations is only slightly shifted towards the 3′-fold conformation (*K*^3^′^/5^′^+3eq. StpA-CTD^_10°C_ = 6.2 ± 0.4 to *K*^3^′^/5^′^+3eq. StpA-CTD^_40°C_ = 3.9 ± 0.4) (SI Supplementary Table S7). The difference in the energy separation is in the same order of magnitude (Δ*G*^3^′^/5^′^+3eq. StpACTD^_25°C_ = −3.75 ± 0.62 kJ/mol). The effect of the chaperone on the RNA equilibrium interestingly levels off at three equivalents StpA-CTD per equivalent RNA (Figure 2C, SI Supplementary Figure S10). The free energy Δ*G* of the refolding process is decreased by StpA-CTD at higher temperatures, whereas the influence can be neglected at temperatures lower than 15°C (Figure 2D, SI Supplementary Table S8). This means in turn that the temperature effect on the RNA equilibrium is much more reduced resulting in a much flatter gradient of Δ*G*/dT^3^′^/5^′^+3.eq StpACTD^ = 27 ± 5 J/mol K.

### Thermodynamics of StpA-binding

**Figure 3.**
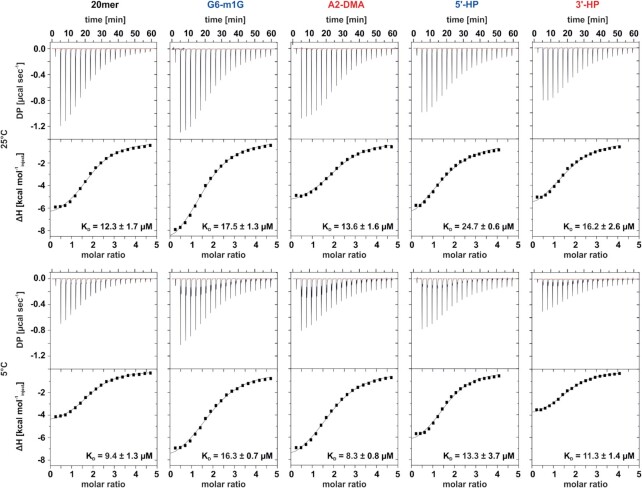
ITC thermograms and fits for StpA binding to the bistable 20mer RNA, the trapped 20mer constructs (G6-m1G, A2-DMA) and the hairpin conformations (5′-HP, 3′-HP) each RNA 40 μM at 5°C and 25°C, StpA 800 μM (3′-HP, 5′-HP)/ 920 μM (bistable 20mer, A2-DMA, G6-m1G). All ITC-experiments shown here were carried out in a buffer containing 50 mM BisTris, 25 mM NaCl and pH 6.4. Errors represent the standard deviation of three independent measurements.

The thermodynamics of the binding of StpA to the 20 nt RNA was characterized by ITC measurements gaining *K*_D_ values as well as the thermodynamic signatures of the binding at 5°C and 25°C. Supplementary to the bistable RNA sequence we utilize another five RNAs to obtain insight into the two single conformations and the role of the single strand overhangs, that are at least predicted to be hubs for the interaction between chaperones and RNA (15). The additional sequences are the respective truncated hairpin structures of the 5′-fold and the 3′-fold conformations as well as chemically modified RNAs that are conformationally locked. The latter ones (see Figure 5E) contain methyl groups that replace hydrogens of the Watson-Crick interface and inhibit the formation of alternative base pairs needed for refolding. For the 5′-fold a methyl group at position N1 of the nucleobase of guanosine G6 (G6-m1G) is incorporated and for the 3′-fold, two methyl groups replace the amino protons in nucleotide A2 (A2-DMA). Further, the 7 nt long single stranded overhang of the 5′-fold (5′-SS OV) was used for the binding studies. For the structured RNAs, the dissociation constants (*K*_D_) range from 12.3 to 24.7 μM at 25°C and from 8.3 to 16.3 μM at 5°C and show pronounced trends (see SI Supplementary Table S9 and SI Supplementary Figure S12). For each construct the affinity increases with lower temperature (Figure 3). Generally, the full length RNAs exhibit tighter binding than the truncated model structures. Within the 3′- and 5′-conformations, the full length RNAs show a better affinity than the corresponding hairpin structures, except for the 5′HP at 5°C. By comparing the two conformational sides, the 3′-conformation shows lower *K*_D_ values than the 5′-conformations. StpA seems to have a higher affinity toward the 3′-conformation of the bistable RNA and the single strand overhang has an increasing effect onto the affinity. The latter might be due to increased flexibility and therefore decreased stability. However, the 7 nt long overhang alone shows virtually no binding at 25°C and only minute response at 5°C (see SI Supplementary Figure S13B). This might indicate a very weak affinity of StpA for short single stranded sequences or that in this case binding occurs with no recalescence. For all constructs that show a proper response in the ITC measurements, the hill coefficient is around n = 2, indicating a cooperative binding. In depth analysis of the ITC data by thermodynamic signature plots (44–46) reveals that for all RNAs the binding is exothermic and enthalpy driven (SI Supplementary Figure S14). Further, it becomes obvious that the RNAs from the 3′-conformational side show a different signature in comparison to those from the 5′-conformationals side. Whereas –*T*Δ*S* is always positive for 5′-side RNAs, it is almost always negative for those RNAs representing the 3′-side of the equilibrium. This finding indicates a more balanced binding based on hydrophobic effects as well as hydrogen and van der Waals bonds present for the 3′-side RNAs. Consequently, there is a different mode of binding of StpA to 3′- and 5′-conformations regarding the 20 nt RNA and also a conformation dependent affinity trend.

### Real-time NMR

**Figure 4.**
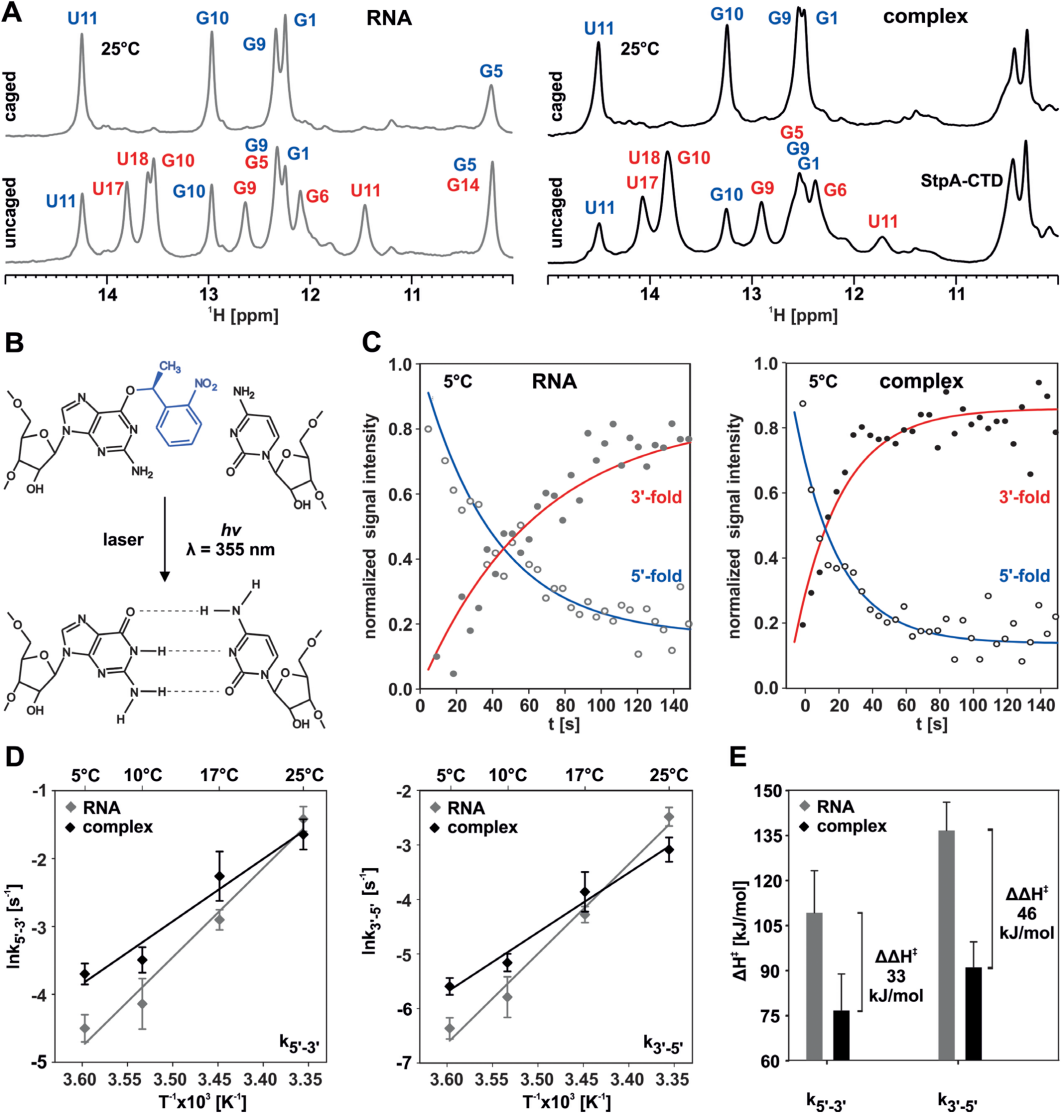
(**A**) ^1^H NMR spectra of imino protons of the 20 nt RNA alone (left panel) and in complex with three equivalents of the RNA chaperone StpA-CTD (right panel), (**B**) caged: *O*^6^-(*S*)-NPE modified guanosine at position G6, uncaged: same samples after photolysis by a laser pulse (λ = 355 nm, *P* = 4 W, *t* = 1 s) after 30 min of equilibration at 25°C. (B) (*S*)-NPE (blue) caged guanosine and cytosine that are not able to base pair because of steric hindrance. Photolytic cleavage with laser light at 355 nm and subsequent formation of GC base pair. (**C**) Normalized imino proton signal intensities (U17 3′-fold, U11 5′-fold) as function of time at 10°C. *k*_5′-3′RNA_ = (16.6 ± 6)10^–3^ s^–1^, *k*_3′-5′RNA_ = (3.1 ± 1.2)10^–3^ s^–1^, *k*_5′-3′complex_ = (30.4 ± 5)10^–3^ s^–1^, *k*_3′-5′complex_ = (4.9 ± 0.9)10^–3^ s^–1^. Error represent the standard deviation of the rate constants for individual peaks. (**D**) Arrhenius plot of *k*_5′-3′_ and *k*_3′-5′_ for the RNA (gray) and the RNA chaperone complex (black). Error bars represent the standard deviation of the rate constants for individual peaks E) Activation enthalpy for the refolding reaction Δ*H*^‡^ for the RNA (gray) and the complex. (black) calculated by with ln(*k*) = Δ*H*^‡^/(*RT*) + ln *A* from the *k*_5′-3′_ and *k*_3′-5′_ rates. Regarding (D) error bars represent the standard deviation for the mean values of the exchange rates. Regarding (**E**) error bars represent the error of the Arrhenius fit. Concentration of the RNA in each experiment c(RNA) = 100 μM.

To probe the changes in the refolding kinetics of the bistable RNA in the presence of StpA-CTD, we utilized photocaged RNAs in conjunction with laser-assisted real-time NMR experiments (8). This methodology allows following the RNA refolding kinetics at atomic resolution. By incorporation of a bulky and photo-labile NPE group at position of guanosine 6, the less stable 5′-fold should be exclusively populated. This is because in the 5′-fold the incorporation at the loop position is structurally silent, whereas in the 3′-fold the modification induces a selective destabilization of the Watson−Crick G6C19 base pair. By NMR, we can unambiguously show that indeed only the 5′-fold is exclusively populated, as no signals stemming from the 3′-fold but all corresponding five imino signals of the 5′-fold are observable in the proton NMR spectra (Figure 4A). Also, in presence of StpA-CTD only the signals of the 5′-fold are detectable, however due to the complex formation the linewidth is increased. Photolytic cleavage of the NPE group by *in situ* uncaging inside the spectrometer with a laser pulse restores the base pairing properties of the guanosine (Figure 4B). Starting from the preselected conformational state, the subsequent refolding into the equilibrium can take place. After equilibration of the samples, all imino signals both of the 5′-fold and the 3′-fold are present as in the parental RNA sequence. This indicates that both for the RNA alone as well as in the presence of StpA-CTD, the deprotection of the G6-NPE occurred fast and nearly to completion (88%) (SI Supplementary Table S10). If the *in-situ* laser-deprotection of the caged bistable RNA is coordinated with a pseudo-2D ^1^H-jump-return experiment (47) kinetic traces reporting on peaks of the 3′- and 5′-fold can be extracted (Figure 4C). Both in absence and presence of StpA-CTD, kinetic traces of all peaks can best be fitted to a unimolecular two-state model. Beyond experimental error, no nucleotide specific difference in the refolding kinetics can be detected. The two-state model is further corroborated by the fact that no additional peaks reporting on an intermediate state are observable. For the RNA without StpA-CTD, the mean rates for the forward reaction *k*_5′-3′_ are (11 ± 3) × 10^–3^ s^−1^ and (240 ± 40) × 10^–3^ s^−1^ at 5°C and 25°C, respectively. The rate of the backward reaction *k*_3′-5′_ is determined to be (1.7 ± 0.3) × 10^–3^ s^−1^ to (84 ± 15) × 10^–3^ s^−1^ at 5°C and 25°C. In the presence of 3 eq. of StpA-CTD *k*_5′-3′_ ranges from (25 ± 3) × 10^–3^ s^-1^ to (190 ± 40) × 10^-3^ s^−1^ and *k*_3′-5′_ (3.7 ± 0.6) × 10^–3^ s^−1^ to (46 ± 10) × 10^–3^ s^−1^ (see SI Supplementary Tables S11 and S12). From these data, we find that at low temperatures StpA-CTD accelerates the refolding in both directions up to a factor of ∼2. However, at higher temperatures, the effect of StpA-CTD on the corresponding refolding kinetics is negligible (Figure 4D). When kinetic data at various temperatures are translated into transition state energies, we find in the presence of the chaperone a substantial decrease of the activation enthalpy ΔH^‡^ of the refolding by 33 kJ/mol (for 5′-3′ transition) and 46 kJ/mol (for 3′-5′ transition) (Figure 4E). This decrease implies that in the presence of StpA-CTD the energy barrier of the refolding reaction is reduced and consequently that less energy is required to disrupt the favorable base-pair interactions in the respective initial conformational state of the refolding reaction. In order to exclude that these findings are only due to molecular crowding through the protein, refolding experiments were conducted in the presence of a non-proteinaceous crowding agent. The refolding of the bistable RNAs was probed again by real-time NMR experiments but now in the presence of 8% (w/v) PEG-8000 at 5°C and 25°C (48,49). As verified through analysis of the imino and aromatic signals in the proton 1D NMR spectra (see SI Supplementary Figure S16) PEG-8000, in comparison to StpA, is not binding to the caged RNA as well as to the uncaged RNA. However, at 5°C PEG-8000 exhibits a minute accelerating effect on the refolding by a factor of 1.39 ± 0.39 (*k*_5′-3′_ and *k*_3′-5′_) (see SI Supplementary Tables S13 and S14). In contrast, StpA shows a significant higher acceleration of 2.17 ± 0.43 (*k*_5′-3′_) and 2.21 ± 0.44 (*k*_3′-5′_). The kinetic results at 25°C indicate, that the activity of the chaperone is much more temperature dependent, than the influence of the molecular crowder PEG-8000 (see SI Supplementary Table S15). Consequently, the influence of StpA on the kinetics of the RNA refolding can not only be explained by effects of molecular crowing, but must result from additional molecular interactions that are defining its chaperone activity.

### Base pair stabilities

**Figure 5.**
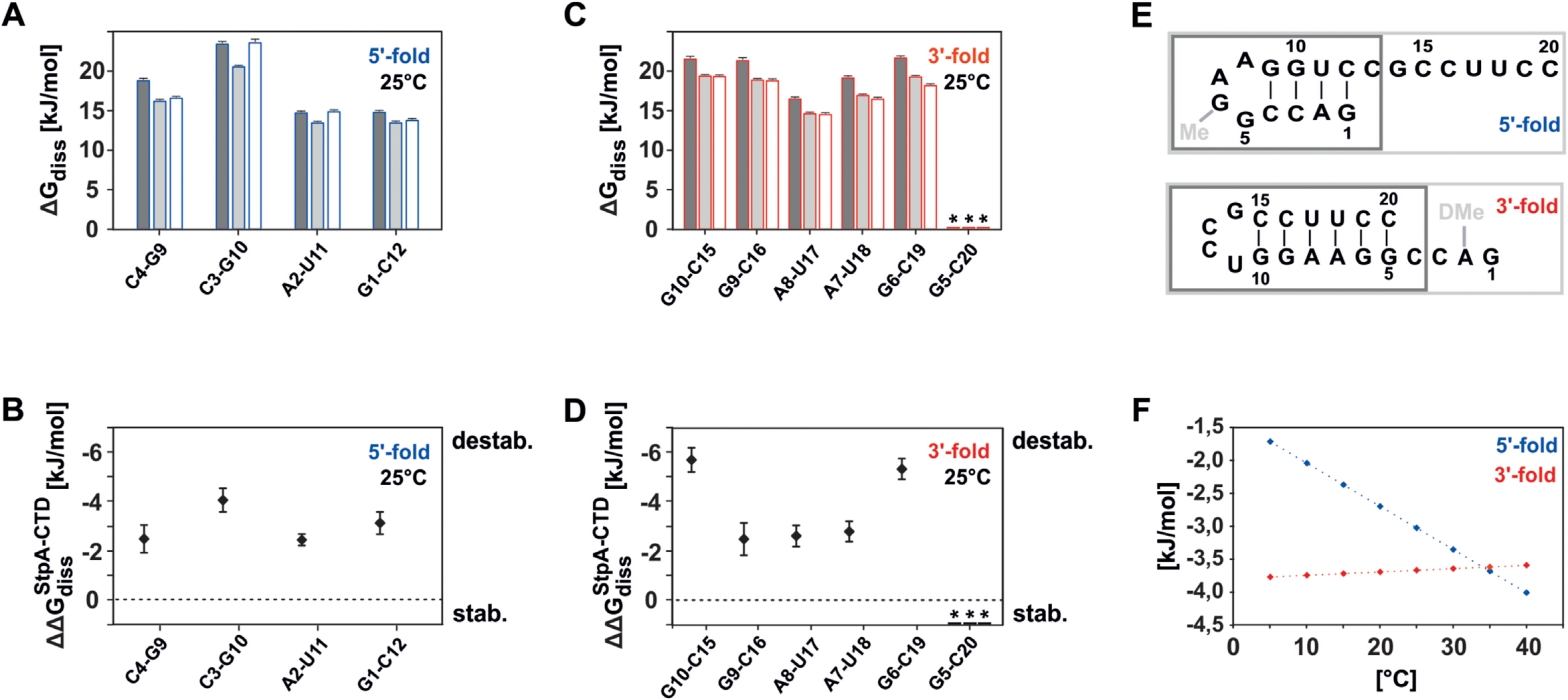
(**A**, **C**) Plots of base pair stabilities in the 5′- (blue) and 3′-fold (red) by means of dissociation free energy Δ*G*_diss_ (*T* = 25°C) for the different RNAs constructs, error bars represent the standard deviation, calculated from the confidence interval of *k*_ex_(*T*) color code: dark gray unmodified RNA, light gray G6-m1G/ A2-DMA RNA, white 5′-HP/3′-HP truncated RNA; (**B**, **D**) Decrease of base pair stability ΔΔ*G*_diss_ for the base pairs in the unmodified RNA constructs upon complex formation with StpA-CTD, defined as ΔΔ*G*_diss_ = Δ*G*_diss_(RNA alone) – Δ*G*_diss_(RNA in complex with three equivalents of StpA-CTD). (**E**) Schematic overview of the different RNA structures of the 5′- and 3′-fold investigated with base pair exchange experiments. (**F**) Temperature dependence of the mean destabilization ΔΔ*G*_diss_ by StpA-CTD. (blue) Reduction of base pair stability within the 5′-fold, (red) reduction of base pair stability within the 3′-fold.* The imino signal of the base pair G5-C20 is broadened beyond detection limit for the water exchange experiment, especially at high temperatures. Concentration of the RNAs given in SI (Supplementary Table S6).

In order to evaluate the influence of StpA’s interaction on the local stability of the RNA, the energetic contribution of each base pair interaction to the refolding reaction and its modulation through the chaperone, we examined the local stability by means of temperature-dependent solvent exchange rates (6,50,51) (see SI Figure S18). For these experiments the same set of RNA constructs as utilized for ITC measurements were used, namely the unmodified 20mer RNA, the full length trapped conformations (G6-m1G, A2-DMA) and the truncated hairpins (5′-HP, 3′-HP) (see Figure 5E). The four additional RNA constructs show only the expected set of imino signal corresponding to their predicted secondary structure (SI Supplementary Figure S17). For all samples the base pair stability was measured as described earlier (6,50). For the RNAs alone we observe that the 3′-fold of the RNA shows a more uniform stability for all base pairs than the 5′-fold (Figure 5A and C). Within the stem of the 3′-fold the lowest stability is measured for the stack of the AU base pairs U17A8 and U18A7. In the 5′-fold stability decreases towards the helical ends and the base pair C3G10 with the highest stability resides in the middle of the helical structure. On average the base pairs in the 3′-folds are more stable than the ones in the 5′-folds. The addition of the single stranded overhangs does not change the stabilities within the hairpin structures significantly. Upon addition of three equivalents of StpA-CTD to the unmodified RNA peaks from the loops are broadened beyond detectability. These are the signals of the imino protons of U11 and G14 from the YNMG-loop capping the 3′-fold and G5 of the GNRA-loop from the 5′-fold (see SI Supplementary Figure S17). While all base pairs exhibit a significant reduction of ΔG_diss_ the overall trend within the helical stems remains the same (Figure 5B and D, Table 1), from which it can be concluded that in presence of StpA the refolding mechanism stays the same. On average, the 5′-fold stays less stable than the 3′-fold. In the 5′-fold the stability of G1 and G10 is reduced most, and in the 3′-fold the nucleotides G10 and G6 show the strongest decrease in dissociation energy (see SI Supplementary Tables S16 – S21). In summary, we find that StpA-CTD induces a selective destabilization of base pairs in proximity of the fraying ends and terminal loops in both conformations (see SI Supplementary Table S22).

**Table 1.** Δ*H*_diss_, Δ*S*_diss_*T_,_ Δ*G*_diss_ at *T* = 25°C for the base pair opening of individual nucleobases within the unmodified 3′-fold and 5′-fold RNA alone and in complex with three eq. of StpA-CTD. Errors represent the standard deviation, calculated from the confidence interval of kex (*T*). Concentration of the RNAs given in SI (Supplementary Table S6)

base pairs		Δ*H*_diss_ (kJ/mol)		Δ*S*_diss_*T (kJ/mol)		Δ*G*_diss_ (kJ/mol)	
		RNA	Complex	RNA	Complex	RNA	Complex
**5**′**-fold**	**C4-G9**	16.69 ± 0.11	22.80 ± 4.09	0.00 ± 0.00	8.60 ± 4.49	16.69 ± 0.12	14.21 ± 0.45
	**C3-G10**	93.23 ± 1.59	101.49 ± 9.54	69.80 ± 1.41	82.11 ± 0.48	23.43 ± 0.20	19.38 ± 0.45
	**A2-U11**	35.30 ± 1.12	80.52 ± 12.17	69.06 ± 4.01	68.24 ± 12.40	14.72 ± 0.09	12.28 ± 0.23
	**G1-C12**	35.34 ± 1.18	41.78 ± 7.14	20.53 ± 1.27	30.09 ± 7.64	14.81 ± 0.10	11.69 ± 0.56
**3**′**-fold**	**G10-C15**	56.82 ± 2.11	47.86 ± 0.51	35.29 ± 1.90	32.05 ± 0.93	21.53 ± 0.28	15.82 ± 0.39
	**G9-C16**	46.10 ± 2.36	27.48 ± 3.86	24.75 ± 2.14	8.62 ± 3.30	21.35 ± 0.29	18.86 ± 0.60
	**A8-U17**	36.83 ± 0.45	22.52 ± 3.16	20.35 ± 0.54	8.65 ± 3.55	16.49 ± 0.10	13.87 ± 0.27
	**A7-U18**	58.41 ± 0.11	62.44 ± 2.41	39.25 ± 0.21	46.08 ± 2.88	19.15 ± 0.11	16.35 ± 0.36
	**G6-C19**	70.57 ± 0.32	66.50 ± 3.92	48.90 ± 0.19	50.16 ± 4.29	1.67 ± 0.13	16.33 ± 0.36

## DISCUSSION

**Figure 6.**
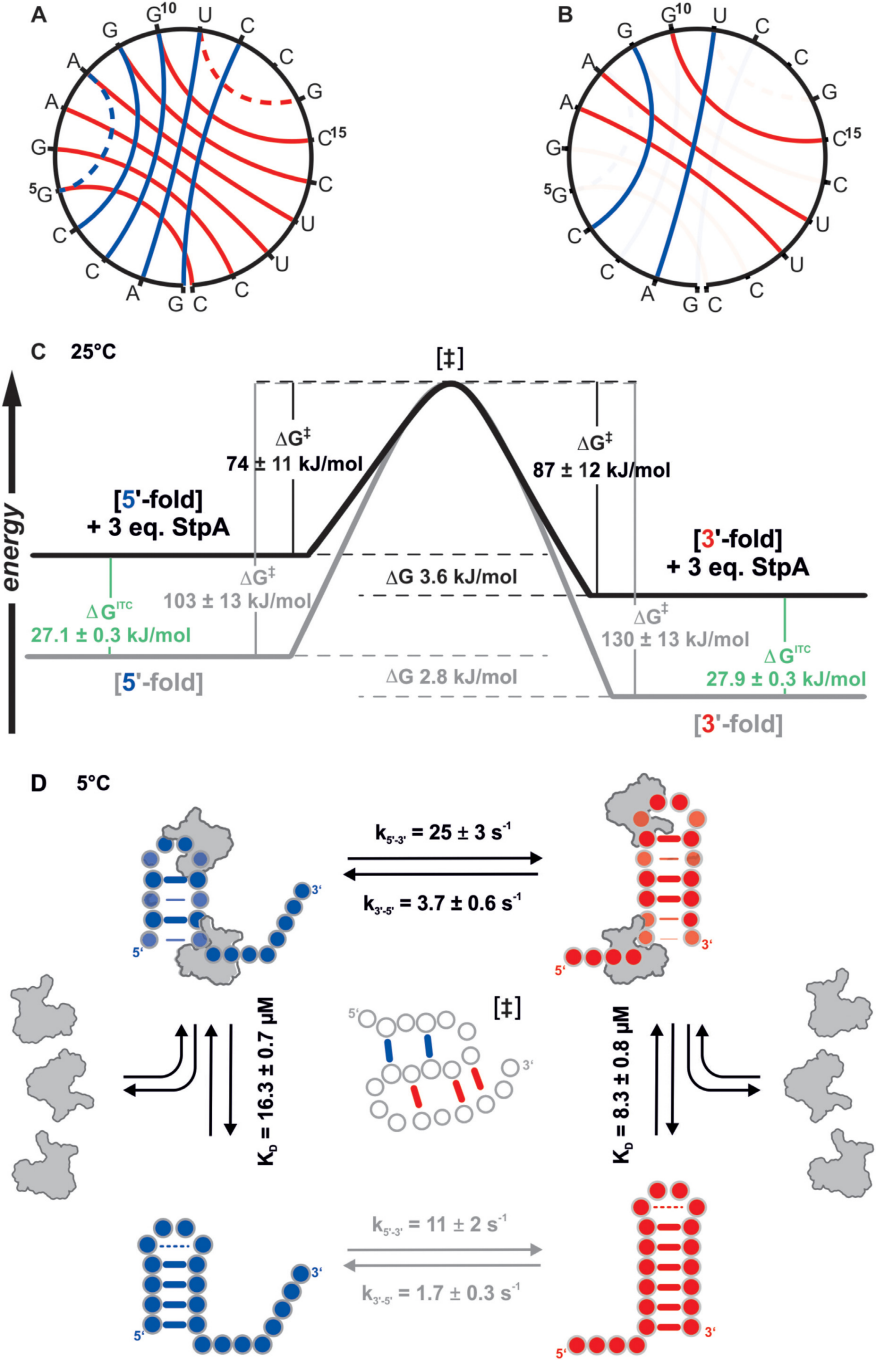
(**A**) Circular structure plots representing the base pairing interactions in 5′- and 3′-fold in blue and red, respectively, dotted lines represent non canonical base pairing interactions in the loops. (**B**) Circular structure plots representing the base pairing interactions in 5′- and 3′-fold in complex with StpA-CTD, base pairing interactions that are destabilized by more than ΔΔ*G*_diss_^StpA-CTD^ > 3 kJ/mol are shaded. (**C**) Energy diagram of the refolding process for the RNA alone (gray) and in complex with StpA-CTD (black); values of Δ*G*^‡^ (derived from an Eyring analysis), Δ*G*^ITC^ (derived from ITC measurements for A2-DMA, G6-m1G) and Δ*G* are given for *T* = 25°C. (**D**) Conformational equilibrium of bistable 20mer RNA and binding of StpA including the rate constants (*k*_5′-3′_ and *k*_3′-5′_) and dissociation constants *K*_D_ for *T* = 5°C.

Generally, refolding of RNAs between stable conformations can occur on different pathways, dependent on the exact sequence context. One of the most prevalent mechanisms is the refolding through a pseudoknotted transition state (12,47). The 20 nt bistable RNA examined here also favors this pathway over a pathway featured by a fully unfolded transition state. This is highlighted by the fact that the activation enthalpy corresponds to half of the entire hairpin enthalpy (12). Here, we can now show that the RNA chaperone StpA-CTD, that is known to bind weakly and transiently to RNA (28), binds with a *K*_D_ in the lower decadic μM range and promotes the refolding reaction by modulation of the underlying thermodynamics of the bistable system. Transient complex formation leads to a selective destabilization of base pairs that translates into differential destabilization of the ground state conformations. With increasing temperature, the equilibrium between the 5′-fold and 3′-fold is shifted towards the overall more stable conformation of the 3′-fold. This is also in line with the finding that the affinity of StpA for the 3′-fold is slightly higher than for the 5′-fold. The reason for this behavior sources in the StpA-CTD dependent modulation of local base pair stability. The reduction of the base pair stability is almost temperature-independent for the 3′-fold, whereas the destabilization within the 5′-fold increases significantly within the temperature range from 5°C to 40°C (Figure 5F). The selective destabilization of the mutually exclusive base pairs of the two conformations assists the formation of a pseudoknotted transition state with five base paring interactions. This is in line with the finding that this RNA predominantly refolds through such a pseudoknotted conformation and that in the transition state of bistable RNAs half of the ground state interactions is retained (Figure 6) (8,12). Based on the modulation of base-pair stabilities and the fact that StpA has a uniform effect on the chemical shifts of the RNA, the other refolding mechanisms can be regarded as insignificant for this bistable system. The selective destabilization is the first step of the pseudo-knotted refolding mechanism and leads to an accelerated refolding as shown in the kinetic results. At low temperatures the refolding kinetics of the model RNA are increased by factor of ∼2 and we could exclude that this is only due to molecular crowding, but to the specific chaperone activity of StpA. The 2-fold StpA induced increase in refolding rates is in line with earlier findings where the RNA chaperone activity was probed in cis-splicing assays. In these assays the observable reaction rate was increased by factors of 1.1–1.3 (28) and 1.4 and 5 (52). However, in these assays the ratio of chaperone over RNA is 10^6^-fold higher than in the real-time NMR experiments presented here. In experiments measuring the annealing rate of two single stranded RNAs the acceleration factor for StpA is found to be around 4 (28,53), however such a reaction does not reflect naturally occurring refolding reactions, where the opening of pre-folded base pair interactions represents the time limiting step. *In vivo* a plethora of proteins is present that exhibits RNA chaperone activity (17,26,54). Their activity on resolving misfolded or kinetically trapped RNA conformations could be shown experimentally. However, *in vivo* a direct measurement of the chaperone effect on refolding rates is still missing. Undoubtedly, the ratio of concentration of chaperones over RNAs exceeds the experimental conditions used here by far and will therefore lead to a higher absolute refolding speed. For example, per bacterial cell the amount of a single mRNA is in the order of 1–10 copies, but the protein StpA is expressed up to ∼10000 copies per cell. Further, there are also other proteins expressed that harbor chaperone activity. Therefore, the two-fold acceleration measured here for a ratio of three chaperone molecules per RNA represents the lower limit. Nevertheless, the underlying mechanism of StpA’s chaperone activity will not be changed due to differences in concentrations. Even under *in vivo* conditions, the reduction of ground state stability will consequently lead to a decrease of activation energy by preparing the conformations for the refolding reaction. However, as this effect is temperature-dependent, the acceleration of the refolding kinetics levels off at higher temperatures. This is in accordance with earlier observations that StpA rescues folding and splicing of the wild type td intron significantly only at lower temperatures (52). Further, RNA chaperones from the class of cold shock proteins also exert their function especially at low temperatures as highlighted in studies of CspA (21,55).

Summarizing, the acceleration of refolding is rather based on the selective destabilization of base pair interactions in the long-lived ground states as opposed to a stabilization of the transition state. The latter plays if at all only a minor role, in line with the expectation that the encounter of StpA-CTD with the short-lived transition state is not productive. It remains to be seen, if other RNA chaperones also act as selective ground state lubricants or act with a yet different mechanism.

## Supplementary Material

gkab876_Supplemental_File

## References

[1] Xayaphoummine A. , Vlasnoff V., Harlepp S., Isambert H Encoding folding paths of RNA switches. Nucleic Acids Res. 2007; 35:614–622. 17178750 10.1093/nar/gkl1036 PMC1802593

[2] Mandal M. , Breaker R.R Gene regulation by riboswitches. Nat. Rev. Mol. Cell Biol. 2004; 5:451–463. 15173824 10.1038/nrm1403

[3] Steinert H. , Sochor F., Wacker A., Buck J., Helmling C., Hiller F., Keyhani S., Noeske J., Grimm S., Rudolph M.M. et al. Pausing guides RNA folding to populate transiently stable RNA structures for riboswitch-based transcription regulation. Elife. 2017; 6:e21297. 28541183 10.7554/eLife.21297 PMC5459577

[4] Ottink O.M. , Rampersad S.M., Tessari M., Zaman G.J.R., Heus H.A., Wijmenga S.S Ligand-induced folding of the guanine-sensing riboswitch is controlled by a combined predetermined induced fit mechanism. RNA. 2007; 13:2202–2212. 17959930 10.1261/rna.635307 PMC2080608

[5] Noeske J. , Richter C., Grundl M.A., Nasiri H.R., Schwalbe H., Wöhnert J An intermolecular base triple as the basis of ligand specificity and affinity in the guanine- and adenine-sensing riboswitch RNAs. Proc. Natl. Acad. Sci. U.S.A. 2005; 102:1372–1377. 15665103 10.1073/pnas.0406347102 PMC547832

[6] Rinnenthal J. , Klinkert B., Narberhaus F., Schwalbe H Direct observation of the temperature-induced melting process of the Salmonella fourU RNA thermometer at base-pair resolution. Nucleic Acids Res. 2010; 38:3834–3847. 20211842 10.1093/nar/gkq124 PMC2887971

[7] Lorenz R. , Bernhart S.H., Höner zu Siederdissen C., Tafer H., Flamm C., Stadler P.F., Hofacker I.L ViennaRNA Package 2.0. Algorithms Mol. Biol. 2011; 6:26. 22115189 10.1186/1748-7188-6-26 PMC3319429

[8] Wenter P. , Fürtig B., Hainard A., Schwalbe H., Pitsch S. Kinetics of photoinduced RNA refolding by real-time NMR spectroscopy. Angew. Chem. Int. Ed. 2005; 44:2600–2603. 10.1002/anie.200462724 15782371

[9] Himmelstoß M. , Erharter K., Renard E., Ennifar E., Kreutz C., Micura R. 2′-O-Trifluoromethylated RNA – a powerful modification for RNA chemistry and NMR spectroscopy. Chem. Sci. 2020; 11:11322–11330. 34094374 10.1039/d0sc04520a PMC8162808

[10] Nußbaumer F. , Plangger R., Roeck M., Kreutz C. Aromatic 19 F–13 C TROSY—[ 19 F, 13 C]-pyrimidine labeling for NMR spectroscopy of RNA. Angew. Chemie Int. Ed. 2020; 59:17062–17069. 10.1002/anie.202006577 PMC7540360 32558232

[11] Fürtig B. , Wenter P., Pitsch S., Schwalbe H. Probing mechanism and transition state of RNA refolding. ACS Chem. Biol. 2010; 5:753–765. 20536261 10.1021/cb100025a

[12] Xu X. , Chen S.-J. Kinetic mechanism of conformational switch between bistable RNA hairpins. J. Am. Chem. Soc. 2012; 134:12499–12507. 22765263 10.1021/ja3013819 PMC3427750

[13] Fürtig B. , Buck J., Manoharan V., Bermel W., Jäschke A., Wenter P., Pitsch S., Schwalbe H. Review time-resolved NMR studies of RNA folding. Biopolymers. 2007; 86:360–383. 17595685 10.1002/bip.20761

[14] Thirumalai D. , Woodson S.A. Kinetics of folding of proteins and RNA. Acc. Chem. Res. 1996; 29:433–439.

[15] Woodson S.A. Taming free energy landscapes with RNA chaperones. RNA Biol. 2010; 7:677–686. 21045544 10.4161/rna.7.6.13615 PMC3073327

[16] Cooper S. , Helmstetter C.E. Chromosome replication and the division cycle of Escherichia coli. J. Mol. Biol. 1968; 31:519–540. 4866337 10.1016/0022-2836(68)90425-7

[17] Waldsich C. , Grossberger R., Schroeder R. RNA chaperone StpA loosens interactions of the tertiary structure in the td group I intron in vivo. Genes Dev. 2002; 16:2300–2312. 12208852 10.1101/gad.231302 PMC186668

[18] Herschlag D. RNA chaperones and the RNA folding problem. J. Biol. Chem. 1995; 270:20871–20874. 7545662 10.1074/jbc.270.36.20871

[19] Doetsch M. , Schroeder R., Fürtig B. Transient RNA-protein interactions in RNA folding. FEBS J. 2011; 278:1634–1642. 21410645 10.1111/j.1742-4658.2011.08094.x PMC3123464

[20] Redder P. , Hausmann S., Khemici V., Yasrebi H., Linder P. Bacterial versatility requires DEAD-box RNA helicases. FEMS Microbiol. Rev. 2015; 39:392–412. 25907111 10.1093/femsre/fuv011

[21] Rennella E. , Sára T., Juen M., Wunderlich C., Imbert L., Solyom Z., Favier A., Ayala I., Weinhäupl K., Schanda P. et al. RNA binding and chaperone activity of the E. coli cold-shock protein CspA. Nucleic Acids Res. 2017; 45:4255–4268. 28126922 10.1093/nar/gkx044 PMC5397153

[22] Shenhar Y. , Biran D., Ron E.Z. Resistance to environmental stress requires the RNA chaperones CspC and CspE. Environ. Microbiol. Rep. 2012; 4:532–539. 23760898 10.1111/j.1758-2229.2012.00358.x

[23] Moll I. , Leitsch D., Steinhauser T., Bläsi U. RNA chaperone activity of the Sm-like Hfq protein. EMBO Rep. 2003; 4:284–289. 12634847 10.1038/sj.embor.embor772 PMC1315900

[24] Arthur D.C. , Ghetu A.F., Gubbins M.J., Edwards R.A., Frost L.S., Glover J.N.M. FinO is an RNA chaperone that facilitates sense-antisense RNA interactions. EMBO J. 2003; 22:6346–6355. 14633993 10.1093/emboj/cdg607 PMC291848

[25] Duval M. , Korepanov A., Fuchsbauer O., Fechter P., Haller A., Fabbretti A., Choulier L., Micura R., Klaholz B.P., Romby P. et al. Escherichia coli ribosomal protein S1 unfolds structured mRNAs onto the ribosome for active translation initiation. PLoS Biol. 2013; 11:12–14. 10.1371/journal.pbio.1001731 PMC3858243 24339747

[26] Clodi E. , Semrad K., Schroeder R. Assaying RNA chaperone activity in vivo using a novel RNA folding trap. EMBO J. 1999; 18:3776–3782. 10393192 10.1093/emboj/18.13.3776 PMC1171454

[27] Rajkowitsch L. , Schroeder R. Dissecting RNA chaperone activity. RNA. 2007; 13:2053–2060. 17901153 10.1261/rna.671807 PMC2080586

[28] Mayer O. , Rajkowitsch L., Lorenz C., Konrat R., Schroeder R. RNA chaperone activity and RNA-binding properties of the *E. coli* protein StpA. Nucleic Acids Res. 2007; 35:1257–1269. 17267410 10.1093/nar/gkl1143 PMC1851640

[29] Sørensen M.A. , Fricke J., Pedersen S. Ribosomal protein S1 is required for translation of most, if not all, natural mRNAs in Escherichia coli in vivo. J. Mol. Biol. 1998; 280:561–569. 9677288 10.1006/jmbi.1998.1909

[30] Sonden B. , Uhlin B.E. Coordinated and differential expression of histone-like proteins in Escherichia coli: regulation and function of the H-NS analog StpA. EMBO J. 1996; 15:4970–4980. 8890170 PMC452234

[31] Woodson S.A. , Panja S., Santiago-Frangos A. Proteins that chaperone RNA regulation. Regulating with RNA in Bacteria and Archaea. 2018; 6:Washington, DC, USA ASM Press 383–397. 10.1128/microbiolspec.rwr-0026-2018 PMC6086601 30051798

[32] Herschlag D. , Khosla M., Tsuchihashi Z., Karpel R.L. An RNA chaperone activity of non-specific RNA binding proteins in hammerhead ribozyme catalysis. EMBO J. 1994; 13:2913–2924. 8026476 10.1002/j.1460-2075.1994.tb06586.x PMC395173

[33] Rajkowitsch L. , Semrad K., Mayer O., Schroeder R. Assays for the RNA chaperone activity of proteins. Biochem. Soc. Trans. 2005; 33:450–456. 15916539 10.1042/BST0330450

[34] Zúñiga S. , Sola I., Cruz J.L.G., Enjuanes L. Role of RNA chaperones in virus replication. Virus Res. 2009; 139:253–266. 18675859 10.1016/j.virusres.2008.06.015 PMC7114511

[35] Tompa P. , Csermely P. The role of structural disorder in the function of RNA and proein chaperones. FASEB J. 2004; 18:1169–1175. 15284216 10.1096/fj.04-1584rev

[36] Doetsch M. , Gstrein T., Schroeder R., Fürtig B. Mechanisms of StpA-mediated RNA remodeling. RNA Biol. 2010; 7:735–743. 21057189 10.4161/rna.7.6.13882 PMC3073332

[37] Flamm C. , Hofacker I.L., Maurer-Stroh S., Stadler P.F., Zehl M. Design of multistable RNA molecules. RNA. 2001; 7:254–265. 11233982 10.1017/s1355838201000863 PMC1370083

[38] Höbartner C. , Ebert M.-O., Jaun B., Micura R. RNA two-state conformation equilibria and the effect of nucleobase methylation. Angew. Chemie Int. Ed. 2002; 41:605–609.

[39] Helmling C. , Wacker A., Wolfinger M.T., Hofacker I.L., Hengesbach M., Fürtig B., Schwalbe H. NMR structural profiling of transcriptional intermediates reveals riboswitch regulation by metastable RNA conformations. J. Am. Chem. Soc. 2017; 139:2647–2656. 28134517 10.1021/jacs.6b10429

[40] Research G. 2020; Deprotection Guide.

[41] MicroCal ITC data analysis in Origin®. Tutor. Guid. 2004; 7:42–43.

[42] Höbartner C. , Micura R. Bistable secondary structures of small RNAs and their structural probing by comparative imino proton NMR spectroscopy. J. Mol. Biol. 2003; 325:421–431. 12498793 10.1016/s0022-2836(02)01243-3

[43] Micura R. , Höbartner C. On secondary structure rearrangements and equilibria of small RNAs. Chem Bio Chem. 2003; 4:984–990. 10.1002/cbic.200300664 14523915

[44] Freire E. Do enthalpy and entropy distinguish first in class from best in class. Drug Discov. Today. 2008; 13:869–874. 18703160 10.1016/j.drudis2008.07.005 PMC2581116

[45] Ohtaka H. , Freire E. Adaptive inhibitors of the HIV-1 protease. Prog. Biophys. Mol. Biol. 2005; 88:193–208. 15572155 10.1016/j.pbiomolbio.2004.07.005

[46] Ruben A.J. , Kiso Y., Freire E. Overcoming roadblocks in lead optimization: a thermodynamic perspective. Chem. Biol. Drug Des. 2006; 67:2–4. 16492143 10.1111/j.1747-0285.2005.00314.x

[47] Fürtig B. , Oberhauser E.M., Zetzsche H., Klötzner D.-P., Heckel A., Schwalbe H. Refolding through a linear transition state enables fast temperature adaptation of a translational riboswitch. Biochemistry. 2020; 59:1081–1086. 32134253 10.1021/acs.biochem.9b01044

[48] Dupuis N.F. , Holmstrom E.D., Nesbitt D.J. Molecular-crowding effects on single-molecule RNA folding/unfolding thermodynamics and kinetics. Proc. Natl. Acad. Sci. 2014; 111:8464–8469. 24850865 10.1073/pnas.1316039111 PMC4060727

[49] Kilburn D. , Roh J.H., Guo L., Briber R.M., Woodson S.A. Molecular crowding stabilizes folded RNA structure by the excluded volume effect. J. Am. Chem. Soc. 2010; 132:8690–8696. 20521820 10.1021/ja101500g PMC2906142

[50] Steinert H.S. , Rinnenthal J., Schwalbe H. Individual basepair stability of DNA and RNA studied by NMR-detected solvent exchange. Biophys. J. 2012; 102:2564–2574. 22713572 10.1016/j.bpj.2012.03.074 PMC3368144

[51] Narberhaus F. , Schwalbe H., Wagner D Mechanistic insights into temperature-dependent regulation of the simple cyanobacterial hsp17 RNA thermometer at base-pair resolution. Nucleic Acids Res. 2015; 43:5572–5585. 25940621 10.1093/nar/gkv414 PMC4477652

[52] Grossberger R. , Mayer O., Waldsich C., Semrad K., Urschitz S., Schroeder R. Influence of RNA structural stability on the RNA chaperone activity of the Escherichia coli protein StpA. Nucleic Acids Res. 2005; 33:2280–2289. 15849314 10.1093/nar/gki515 PMC1084320

[53] Rajkowitsch L. , Schroeder R. Coupling RNA annealing and strand displacement: a FRET-based microplate reader assay for RNA chaperone activity. BioTechniques. 2007; 43:304–310. 17907573 10.2144/000112530

[54] Mahen E.M. , Watson P.Y., Cottrell J.W., Fedor M.J. mRNA secondary structures fold sequentially but exchange rapidly in vivo. PLoS Biol. 2010; 8:e1000307. 20161716 10.1371/journal.pbio.1000307 PMC2817708

[55] Jiang W. , Hou Y., Inouye M. CspA, the major cold-shock protein of *Escherichia coli*, is an RNA chaperone. J. Biol. Chem. 1997; 272:196–202. 8995247 10.1074/jbc.272.1.196

