# Supplementary material for: The RNA chaperone StpA enables fast RNA refolding by destabilization of mutually exclusive base pairs within competing secondary structure elements
Source: Nucleic Acids Res. 2021 Oct 6;49(19):11337–49. doi: 10.1093/nar/gkab876 (PMC8565331; doi:10.1093/nar/gkab876)
Supplement: gkab876_Supplemental_File [file gkab876_supplemental_file.pdf]

The RNA chaperone StpA enables fast RNA refolding by destabilization of mutually exclusive base pairs within competing secondary structure elements

Katharina F. Hohmann[a], Anja Blümler[b], Alexander Heckel[b], and Boris Fürtig*[a]

[a] [b] Goethe University Frankfurt am Main, Max-von-Laue-Strasse 7, 60438 Frankfurt/Main (Germany)

Content

1 Chemical Synthesis 3
1.1 Chemicals and conditions 3
1.2 Synthesis of the (*S*)-NPE caged guanosine ($G^{S\text{-}NPE}$) phosphoramidite 3
1.2.1 Synthesis of 5'-*O*-(4,4'-Dimethoxytrityl)-2'-*O*-triisopropylsilyloxymethyl-N^2-acetyl-O^6-[(*S*)-1-(2-nitrophenyl)ethyl] guanosine (2) 4
1.2.2 Synthesis of 5'-*O*-(4,4'-Dimethoxytrityl)-2'-*O*-triisopropylsilyloxymethyl-O^6-[(*S*)-1-(2-nitrophenyl)ethyl] guanosine (3) 4
1.2.3 Synthesis of 5'-*O*-(4,4'-Dimethoxytrityl)-N^2,3'-*O*-bis(4-isopropylphenoxyacetyl)-2'-*O*-triisopropylsilyloxymethyl-O^6-[(*S*)-1-(2-nitrophenyl)ethyl] guanosine (4) 4
1.2.4 Synthesis of 5'-*O*-(4,4'-Dimethoxytrityl)-N^2-(4-isopropylphenoxyacetyl)-2'-*O*-triisopropylsilyloxymethyl-O^6-[(*S*)-1-(2-nitrophenyl)ethyl] guanosine (5) 5
1.2.5 Synthesis of 5'-*O*-(4,4'-Dimethoxytrityl)-N^2-(4-isopropylphenoxyacetyl)-2'-*O*-triiso-propylsilyloxymethyl-O^6-[(*S*)-1-(2-nitrophenyl)ethyl] guanosine-3'-*O*-(2-cyanoethyl-*N,N*-diisopropyl)phosphoramidite (6) 5
1.3 NMR spectra 6
2 Oligonucleotide synthesis 9
2.1 Mass spectra 9
3 StpA-CTD expression - Sequences 10
4 RNA Sequences, Abbreviations, sample concentration 12
5 NMR spectroscopy 13
6 Data collection and analysis of thermodynamic parameters 14
6.1 Titration 20 nt bistable 14
6.2 Temperature Rows and free energy analysis 15
7 Data collection and analysis real-time NMR 17
7.1 Degree of photolysis 17
7.2 Analysis of uncaged RNA sample 18
7.3 Fit of kinetics rates 18
7.4 Kinetics under molecular Crowding conditions with PEG-8000 19
8 Data collection and analysis of base pairs stabilities by NMR 21

8.1 ^{1}H 1D characterization supplementary RNA constructs 21
8.2 Water exchange rates 21
8.3 Temperature dependence of destabilization induced by StpA-CTD 24
9 ITC isothermal titration calorimetry 16

1 Chemical Synthesis

1.1 Chemicals and conditions

All reactions were performed under argon atmosphere using dry solvents purchased from *Acros Organics* or *Merck KGaA*. Reagents were purchased from *Acros Organics, Merck KGaA, ChemPur, TCI, Alfa Aesar* or *ChemGenes* and used without further purification. For flash chromatography the used silica gel was purchased from *Macherey-Nagel* (particle size: 40-63 µm), solvents were of technical grade. NMR spectra were recorded on *Bruker DPX250, AV400* and *DRX600* instruments at ambient temperature.

1.2 Synthesis of the (*S*)-NPE caged guanosine ($G^{S\text{-}NPE}$) phosphoramidite

The synthesis of the *(S)*-NPE protected guanosine phosphoramidite was performed according to literature procedure (1).

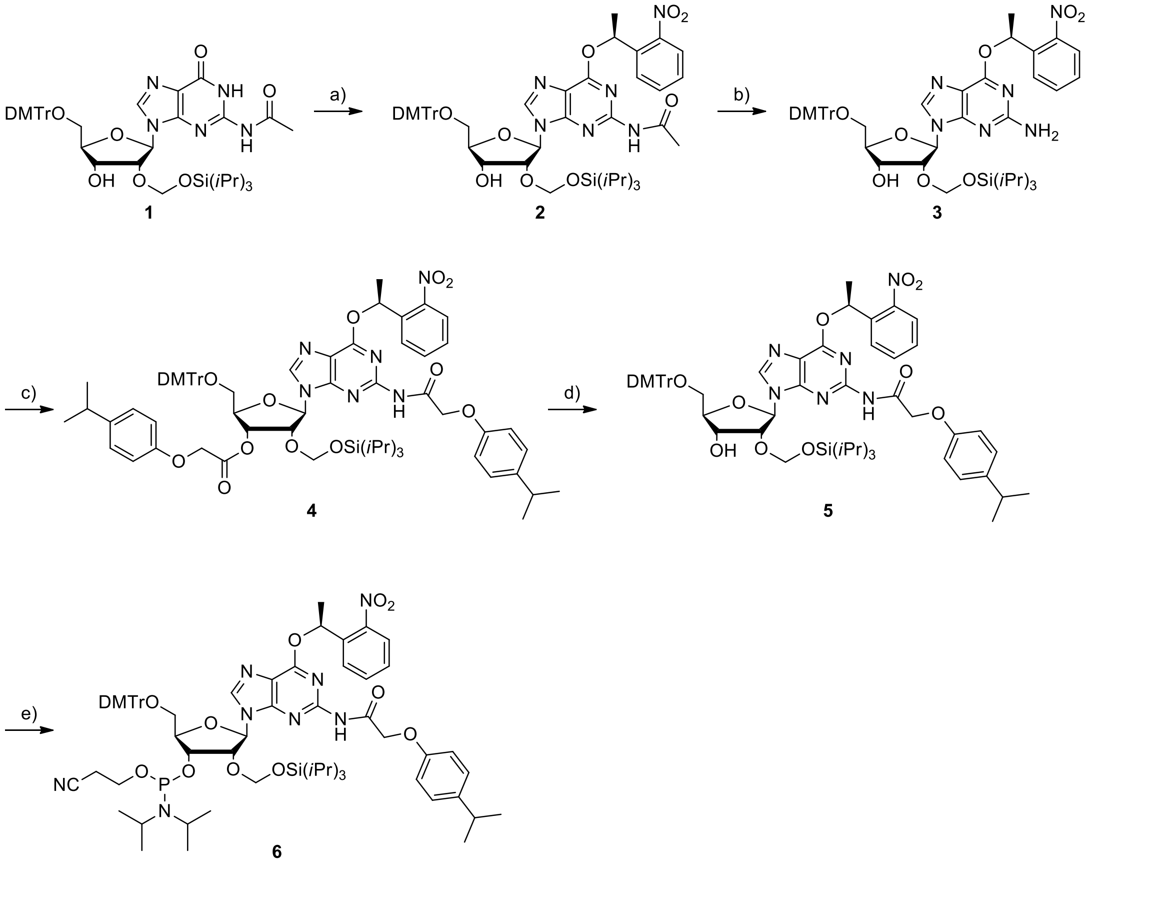

DMTr = 4,4'-dimethoxytrityl

Scheme S1 Overview of the synthesis of $^{(S)\text{-}NPE}$G phosphoramidite **6**. a) **1** (purchased from *ChemGenes*), (*R*)-1-(2-nitrophenyl)ethanol (prepared according to the literature procedure (2)), PPh_3, DIAD, THF, 0°C to rt, 76%; b) 12M $MeNH_2$ in H_2O, THF, rt, 93% (crude product); c) (4-isopropylphenoxy)acetyl chloride, DMAP, pyridine, 0°C to rt, 91%; d) NH_3/MeOH/THF 1:1:1 (v/v/v), rt, 83%; e) 2-cyanoethyl-*N,N*-diisopropylchlorophosphoramidite, DIPEA, CH_2Cl_2, rt, 73%.

1.2.1 Synthesis of 5'-*O*-(4,4'-Dimethoxytrityl)-2'-*O*-triisopropylsilyloxymethyl-N^2-acetyl-O^6-[(*S*)-1-(2-nitrophenyl)ethyl] guanosine (2)

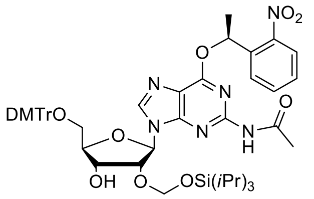

2

1.00 g 5'-*O*-(4,4'-dimethoxytrityl)-2'-*O*-triisopropylsilyloxymethyl-N^2-acetyl guanosine (**1**) (1.23 mmol, 1.0 eq, purchased from *ChemGenes*) was dissolved in 5 mL dry THF. (*R*)-1-(2-nitrophenyl)ethanol(1) (205 mg, 1.23 mmol, 1.0 eq) and PPh_3 (483 mg, 1.84 mmol, 1.5 eq) were added and the resulting solution was cooled with an icebath to 0°C. 363 µL diisopropylazodicarboxylate (DIAD) (1.84 mmol, 1.5 eq) were added dropwise. The reaction mixture was stirred at room temperature for 9 h and concentrated under reduced pressure. The crude product was purified by column chromatography (SiO_2, cyclohexane/EtOAc 4:1+ 1% (v/v) Et_3N → cyclohexane/EtOAc 1:3). Product **2** was isolated as a pale yellowish foam.

Yield: 908 mg (76%)

TLC (cyclohexane/EtOAc 1:1): R_f=0.36

^{1}H-NMR (400 MHz, DMSO-d_6): δ= 10.02 (s, 1H, N*H*-Ac), 8.31 (s, 1H, H8), 8.06-8.04 (m, 1H, $H_{ar,}$ NPE), 7.82-7.80 (m, 1H, $H_{ar,}$ NPE), 7.74-7.70 (m, 1H, $H_{ar,}$ NPE), 7.57-7.53 (m, 1H, $H_{ar,}$ NPE), 7.32-7.30 (m, 2H, $H_{ar,}$ DMTr), 7.21-7.10 (m, 7H, $H_{ar,}$ DMTr), 6.87 (q, ^3J(H,H)=6.5 Hz and 6.3 Hz, 1H, O-C*H*-NPE), 6.81-6.76 (m, 4H, $H_{ar,}$ DMTr), 6.02 (d, ^3J(H,H)=5.6 Hz, 1H, 1'-H), 5.16 (d, ^3J(H,H)=5.7 Hz, 1H, 3'-OH), 4.93-4.92 (m, 1H, O-CH_2-O), 4.89-4.85 (m, 2H, O-CH_2-O and 2'-H), 4.35-4.34 (m, 1H, 3'-H), 4.03-4.02 (m, 1H, 4'-H), 3.70-3.69 (m, 6H, 2x DMTr-OMe), 3.31-3.29 (m, 1H, 5'-H), 3.21-3.18 (m, 1H, 5'-H), 2.08 (s, 3H, NH-Ac), 1.80 (d, ^3J(H,H)=6.5 Hz, 3H, CH_3-NPE), 0.80-0.76 (m, 21H, TOM)ppm.

1.2.2 Synthesis of 5'-*O*-(4,4'-Dimethoxytrityl)-2'-*O*-triisopropylsilyloxymethyl-O^6-[(*S*)-1-(2-nitrophenyl)ethyl] guanosine (3)

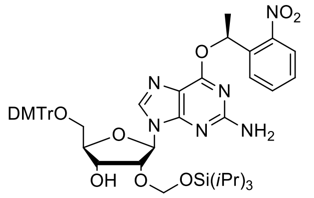

3

850 mg of **2** (881 µmol, 1.0 eq) were dissolved in 8 mL dry THF and treated with 13 mL of 12M $MeNH_2$ in H_2O. After stirring at room temperature for 2 h, the solvent was removed under reduced pressure. The crude product was obtained as a colorless foam and used without further purification in the next synthesis step.

Yield: 758 mg (93%, crude product)

TLC (CH_2Cl_2/acetone 19:1): R_f=0.66

1.2.3 Synthesis of 5'-*O*-(4,4'-Dimethoxytrityl)-N^2,3'-*O*-bis(4-isopropylphenoxyacetyl)-2'-*O*-triisopropylsilyloxymethyl-O^6-[(*S*)-1-(2-nitrophenyl)ethyl] guanosine (4)

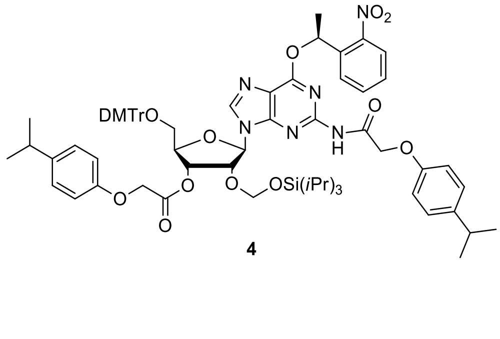

4

A solution of crude **3** (800 mg, 869 µmol, 1.0 eq) and 4-(dimethylamino)pyridine (53 mg, 434 µmol, 0.5 eq) in 4.5 mL pyridine was cooled to 0°C before 751 µL (4-isopropylphenoxy)acetyl chloride (4.34 mmol, 5.0 eq) were added dropwise. The reaction mixture was stirred at room temperature for 18 h. After quenching the reaction by the addition of 40 mL MeOH the solvent was removed under reduced pressure. The crude product was purified by column chromatography (SiO_2, cyclohexane/EtOAc 4:1+ 1% (v/v) Et_3N → cyclohexane/EtOAc 2:3) to give **4** as a pale yellow solid.

Yield: 1.01 g (91%)

TLC (cyclohexane/EtOAc 2:3): R_f=0.73

^{1}H-NMR (400 MHz, DMSO-d_6): δ= 10.09 (s, 1H, N*H*), 8.37 (s, 1H, H8), 8.05-8.03 (m, 1H, $H_{ar,}$ NPE), 7.86-7.83 (m, 1H, $H_{ar,}$ NPE), 7.77-7.72 (m, 1H, $H_{ar,}$ NPE), 7.58-7.54 (m, 1H, $H_{ar,}$ NPE), 7.30-7.28 (m, 2H, $H_{ar,}$ DMTr), 7.20-7.13 (m, 7H, $H_{ar,}$ DMTr), 7.10-7.07 (m, 4H, $H_{ar,}$ *i*PrPac), 6.89-6.87 (m, 1H, O-C*H*-NPE), 6.85-6.82 (m, 4H, $H_{ar,}$ DMTr), 6.80-6.72 (m, 4H, $H_{ar,}$ *i*PrPac), 6.05 (d, ^3J(H,H)=6.8 Hz, 1H, 1'-H), 5.41-5.35 (m, 2H, 2'-H and 3'-H), 4.86-4.63 (m, 6H, O-CH_2-O and 2x CH_2-O*i*PrPac), 4.24-4.22 (m, 1H, 4'-H), 3.68-3.67 (m, 6H, 2x DMTr-OMe), 3.60-3.56 (m, 1H, 5'-H), 3.26-3.22 (m, 1H, 5'-H), 2.86-2.77 (m, 2H, 2x C-*i*PrPac), 1.82 (d, ^3J(H,H)=6.5 Hz, 3H, CH_3-NPE), 1.17-1.12 (m, 12H, CH_3-*i*PrPac), 0.72-0.67 (m, 21H, TOM)ppm.

1.2.4 Synthesis of 5'-*O*-(4,4'-Dimethoxytrityl)-*N*^{2}-(4-isopropylphenoxyacetyl)-2'-*O*-triisopropylsilyloxymethyl-*O*^{6}-[(*S*)-1-(2-nitrophenyl)ethyl] guanosine (5)

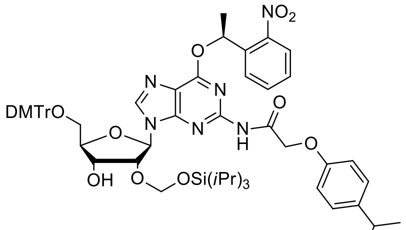

5

1.00 g of **4** (785 µmol, 1.0 eq) were dissolved in 15 mL aq. NH_3/MeOH/THF (1:1:1, v/v) and stirred at room temperature for 2 h. The solvent was removed under reduced pressure and the crude product was purified by column chromatography (SiO_2, cyclohexane/EtOAc 1:1+ 1% (v/v) Et_3N → cyclohexane/EtOAc 1:1) to give **5** as a yellow solid.

Yield: 713 mg (83%)

TLC (cyclohexane/EtOAc 1:1): R_f=0.58

^{1}H-NMR (400 MHz, DMSO-d_6): δ= 10.11 (s, 1H, N*H*), 8.35 (s, 1H, H8), 8.04-8.02 (m, 1H, $H_{ar,}$ NPE), 7.83-7.81 (m, 1H, $H_{ar,}$ NPE), 7.74-7.70 (m, 1H, $H_{ar,}$ NPE), 7.56-7.52 (m, 1H, $H_{ar,}$ NPE), 7.31-7.29 (m, 2H, $H_{ar,}$ DMTr), 7.21-7.10 (m, 9H, 7$H_{ar,}$ DMTr and 2$H_{ar,}$ *i*PrPac), 6.89-6.84 (m, 1H, O-C*H*-NPE), 6.82-6.74 (m, 6H, 4$H_{ar,}$ DMTr and 2$H_{ar,}$ *i*PrPac), 6.05 (d, ^3J(H,H)=5.8 Hz, 1H, 1'-H), 5.17 (d, ^3J(H,H)=5.7 Hz, 1H, 3'-OH), 4.92-4.89 (m, 2H, 2'-H and O-CH_2-O), 4.84-4.83 (m, 1H, O-CH_2-O), 4.77 (brs, 2H, CH_2-O*i*PrPac), 4.32-4.28 (m, 1H, 3'-H), 4.06-4.03 (m, 1H, 4'-H), 3.69-3.68 (m, 6H, 2x DMTr-OMe), 3.38-3.34 (m, 1H, 5'-H), 3.18-3.15 (m, 1H, 5'-H), 2.83 (q, ^3J=5.9 Hz, 1H, C-*i*PrPac), 1.81 (d, ^3J(H,H)=6.4 Hz, 3H, CH_3-NPE), 1.17-1.16 (m, 6H, CH_3-*i*PrPac), 0.76-0.73 (m, 21H, TOM)ppm.

1.2.5 Synthesis of 5'-*O*-(4,4'-Dimethoxytrityl)-*N*^{2}-(4-isopropylphenoxyacetyl)-2'-*O*-triisopropylsilyloxymethyl-*O*^{6}-[(*S*)-1-(2-nitrophenyl)ethyl] guanosine-3'-*O*-(2-cyanoethyl-*N,N*-diisopropyl)phosphoramidite (6)

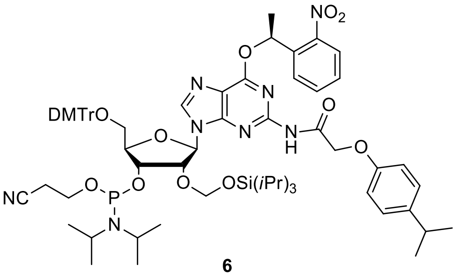

6

500 mg of **5** (456 µmol, 1.0 eq) were dissolved in dry CH_2Cl_2 and treated with 397 µL *N,N*-diisopropylethylamine (2.28 mmol, 5.0 eq). 2-cyanoethyl-*N,N*-diisopropylchlorophosphoramidite (204 µL, 911 µmol, 2.0 eq) was added and the mixture was stirred at room temperature for 6 h. The solution was diluted with 50 mL dry CH_2Cl_2 and washed with saturated aqueous $NaHCO_3$ solution. The organic layer was dried over Na_2SO_4 and the solvent removed under reduced pressure. The crude product was purified by column chromatography (SiO_2, hexane/EtOAc 4:1+ 1% (v/v) Et_3N → hexane/EtOAc 1:3) to give **5** as a slight yellow foam.

Yield: 430 mg (73%)

TLC (cyclohexane/EtOAc 2:1): R_f=0.36

^{1}H-NMR (400 MHz, DMSO-d_6): δ= 10.09-10.6 (m, 1H, N*H*), 8.36 (s, 1H, H8), 8.05-8.03 (m, 1H, $H_{ar,}$ NPE), 7.84-7.82 (m, 1H, $H_{ar,}$ NPE), 7.75-7.71 (m, 1H, $H_{ar,}$ NPE), 7.57-7.53 (m, 1H, $H_{ar,}$ NPE), 7.33-7.28 (m, 2H, $H_{ar,}$ DMTr), 7.23-7.09 (m, 9H, 7$H_{ar,}$ DMTr and 2$H_{ar,}$ *i*PrPac), 6.88-6.86 (m, 1H, O-C*H*-NPE), 6.80-6.72 (m, 6H, 4$H_{ar,}$ DMTr and 2$H_{ar,}$ *i*PrPac), 6.07-6.02 (m, 1H, 1'-H), 5.14-5.07 (m, 1H, 2'-H), 4.89-4.73 (m, 4H, O-C*H*$_2$-O and C*H*$_2$-O*i*PrPac), 4.41-4.34 (m, 1H, 3'-H), 4.18-4.14 (m, 1H, 4'-H), 3.76-3.68 (m, 8H, OC*H*$_2$CH$_2$CN) and 2x DMTr-OMe), 3.58-3.50 (m, 2H, C*H*-*i*Pr), 3.44-3.42 (m, 1H, 5'-H), 3.21-3.18 (m, 1H, 5'-H), 2.83 (m, 1H, COCH$_2$C*H*$_2$CN), 1.81 (d, ^3J(H,H)=6.4 Hz, 3H, C*H*$_3$-NPE), 1.17-1.16 (m, 6H, CH$_3$-*i*Pr), 1.10-1.05 (m, 9H, CH$_3$-*i*Pr), 0.96-0.94 (m, 3H, CH$_3$-*i*Pr), 0.73 (m, 21H, TOM)ppm.

^{31}P-NMR (DMSO-d_6, 162 MHz): δ= 149.55, 149.44ppm.

1.3 NMR spectra

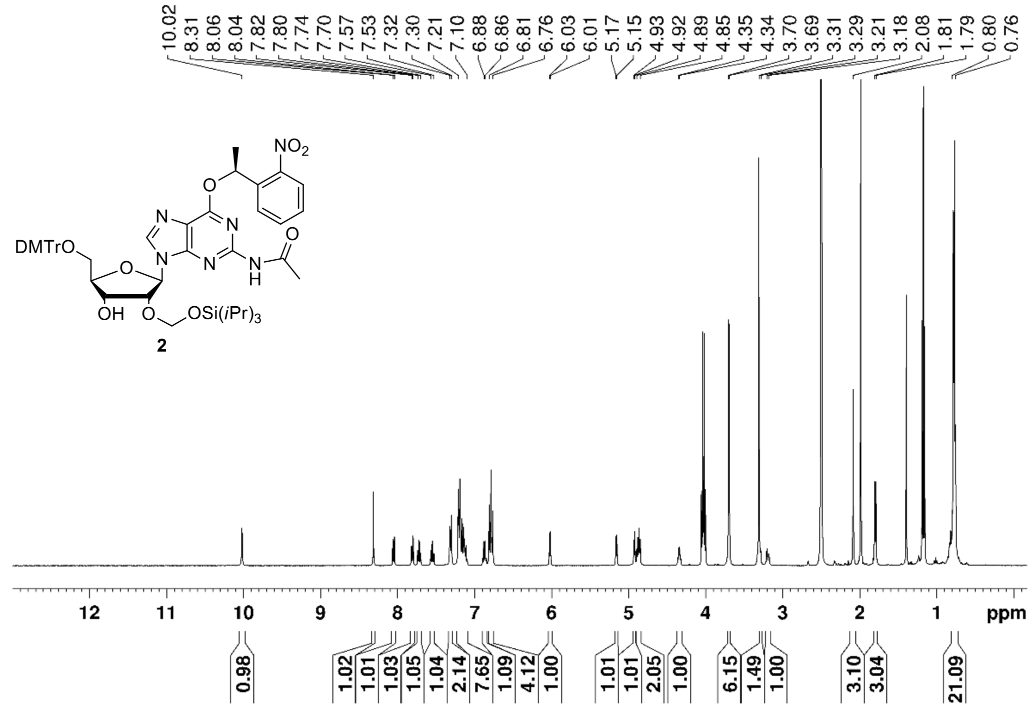

Figure S 1. ^{1}H-NMR spectrum of **2** in DMSO-d_6 (400 MHz, 298 K). Impurities of ethyl acetate and cyclohexane were not assigned.

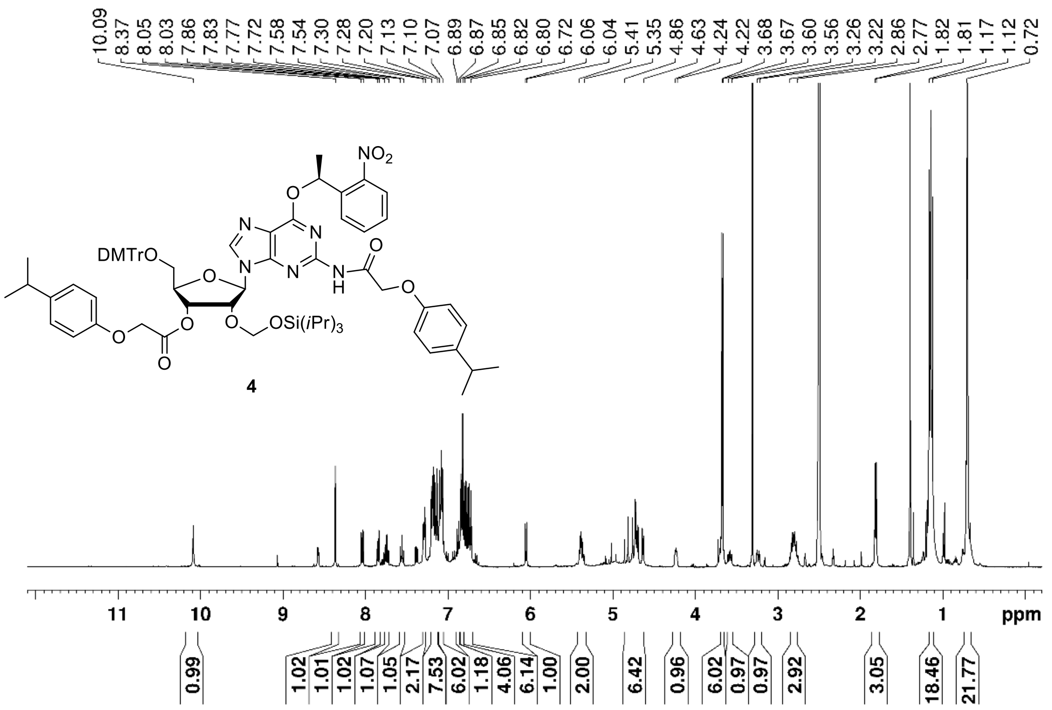

Figure S 2. ^{1}H-NMR spectrum of **4** in DMSO-d_6 (400 MHz, 298 K). Impurities of cyclohexane were not assigned.

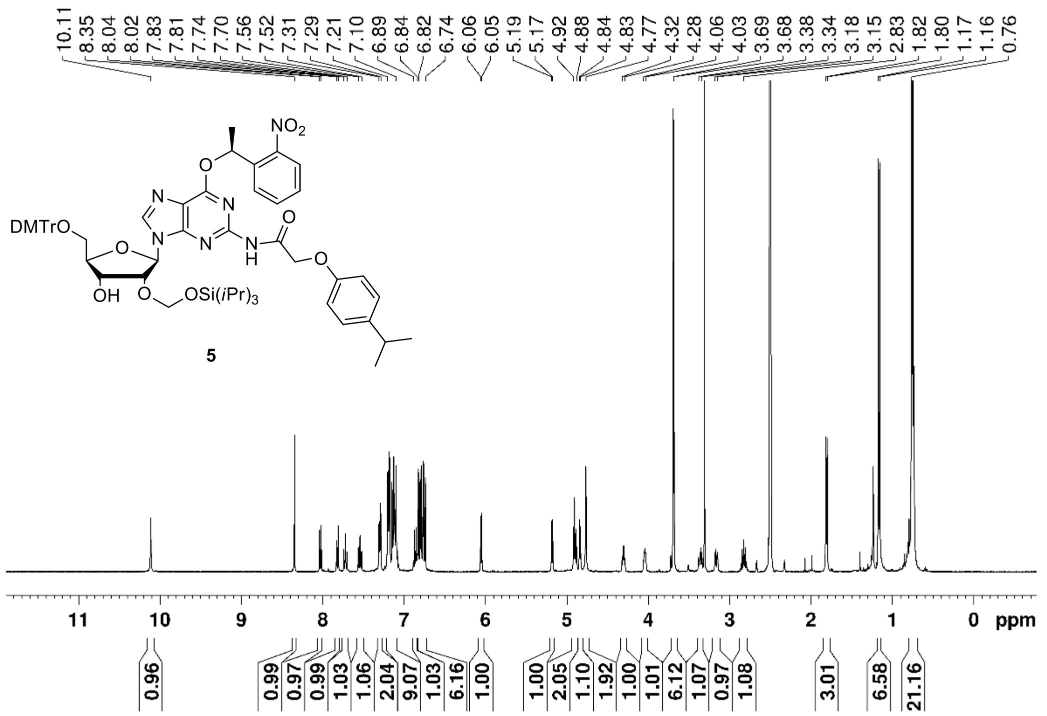

Figure S 3. ^{1}H-NMR spectrum of **5** in DMSO-d_6 (400 MHz, 298 K).

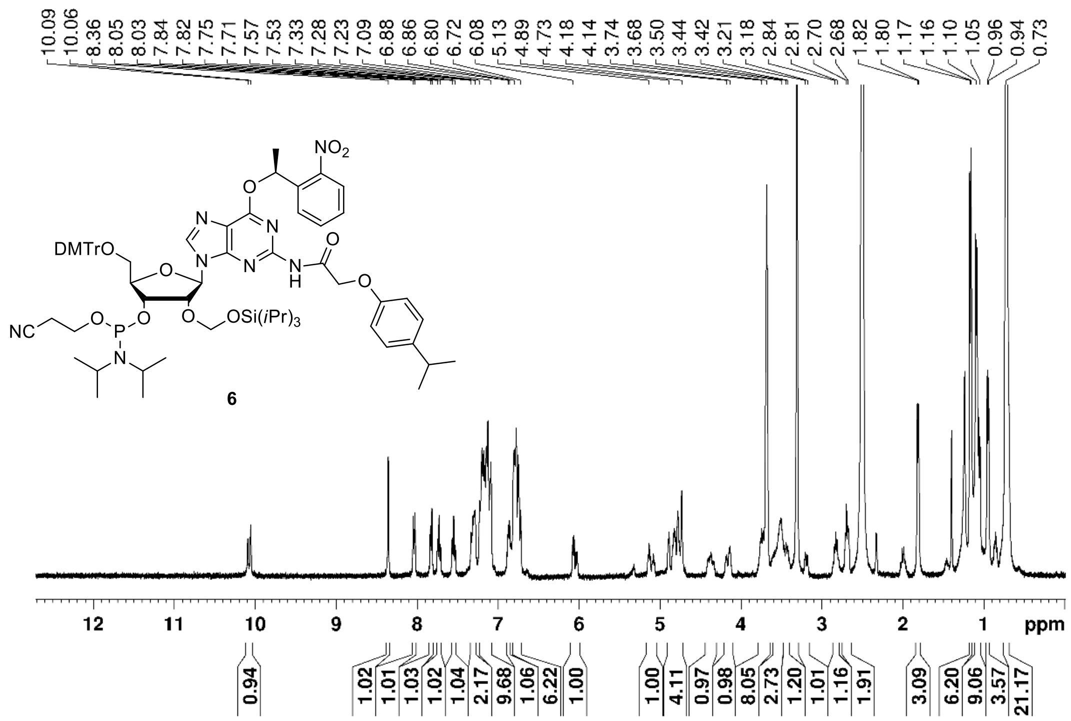

Figure S 4. ^{1}H-NMR spectrum of $^{(S)\text{-NPE}}$rG phosphoramidite **6** in DMSO-d_6 (400 MHz, 298 K).

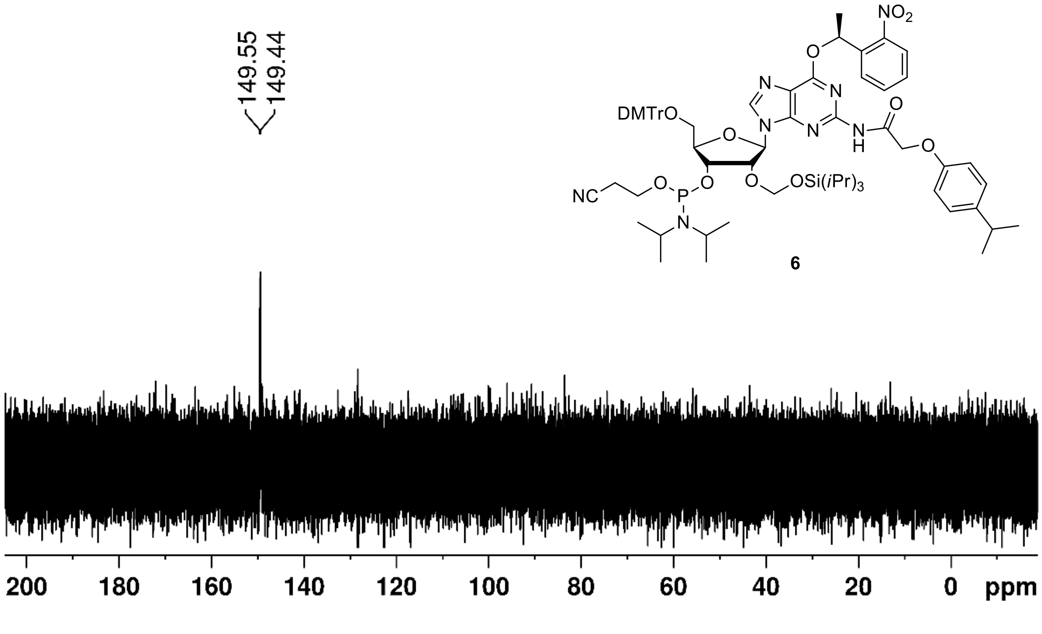

Figure S 5. ^{31}P-NMR spectrum of $^{(S)\text{-NPE}}$rG phosphoramidite **6** in DMSO-d_6 (162 MHz, 298 K).

2 Oligonucleotide synthesis

Table S 1. RP-HPLC conditions used for the RNA purification

time [min]	% solvent A (400 mM HFIP 16.3 mM Et_3N, pH 8.2)	% solvent B (MeOH)
0	95	5
13	76.3	23.7
15	0	100
20	0	100

Table S 2. Sequence and ESI-MS results of the synthesized oligonucleotide

Sequence	Calculated Mass [Da]	Measured Mass [Da]
**RNA:** 5'-r[GAC CG**G**$^{(S\text{-NPE})}$ AAG GUC CGC CUU CC]-3'	6502.9	6503.0

2.1 Mass spectra

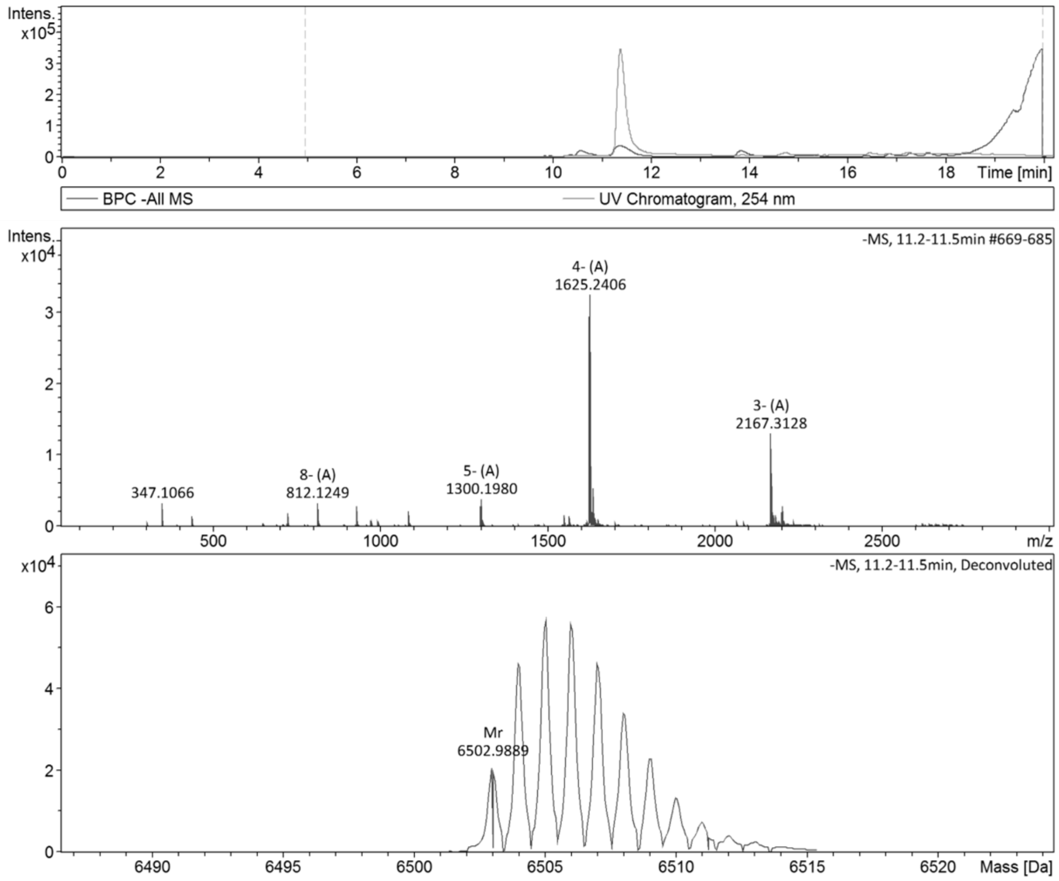

Figure S 6. Mass spectra of photocaged **RNA** recorded on a *Bruker micrOTOF-Q* device (ESI). Top: BPC and UV chromatogram, middle: full spectrum, bottom: deconvoluted molecular peak. The calculated mass is 6502.9.

3 StpA-CTD expression - Sequences

Table S 3. StpA-CTD DNA Sequence that was cloned into the SUMO vector. Primer sequences used for cloning strategy.

StpA-CTD DNA Sequence	1 5'-CGCCAGCCGC GTCCGGCGAA ATATAAATTC ACCGATGTTA<br>51 ACGGTGAAAC TAAAACCTGG ACCGGTCAGG GCCGTACACC<br>91 GAAGCCAATT GCTCAGGCGC TGGCAGAAGG TAAATCTCTC<br>131 GACGATTTCC TGATC-3'
forward primer	5'-CCGGTCTCGAGGTCGCCAGCCGCGTCC-3'
reverse primer	5'-CCGGTCTCTCTAGATTAGATCAGGAAATCGTCGAG AG-3'

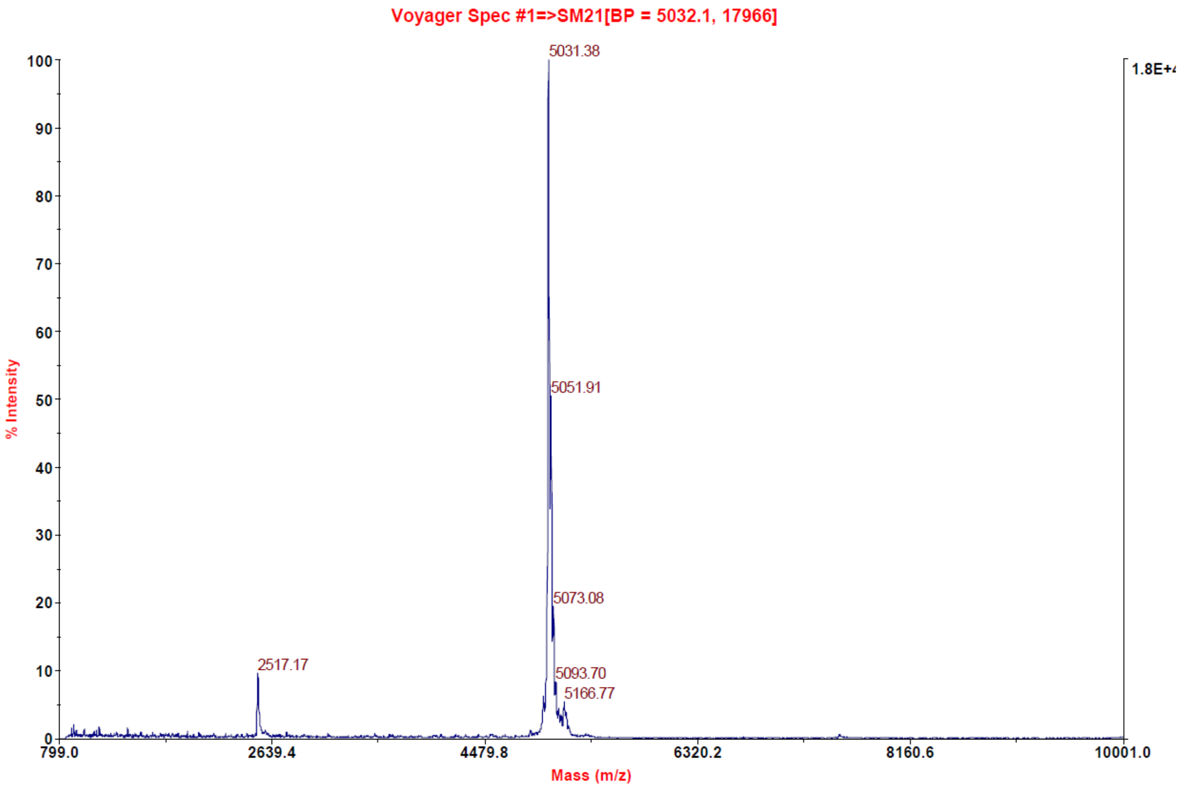

Figure S 7. MALDI Mass spectra of purified StpA-CTD recorded on a Applied Biosystems Voyager-DE STR device, The calculated mass is 5031.71 Da.

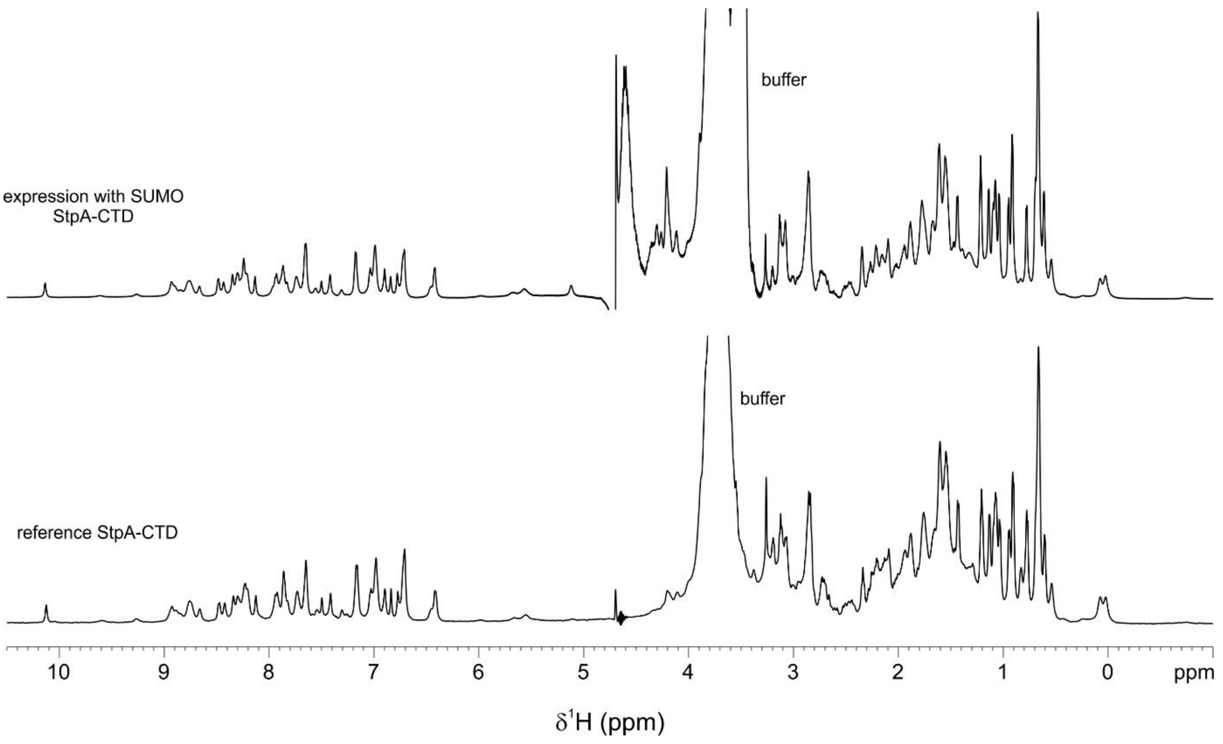

Figure S 8: Amide and aliphatic proton region of ^{1}H NMR spectra of StpA-CTD via expression with SUMOstar vector and of reference StpA-CTD at 25°C, recorded at 800 MHz/ 600 MHz Bruker NMR spectrometers. The expression of StpA-CTD yields correctly folded protein. Concentrations c(expression with SUMO StpA-CTD)= 860 µM, c(reference StpA)=1000 µM.

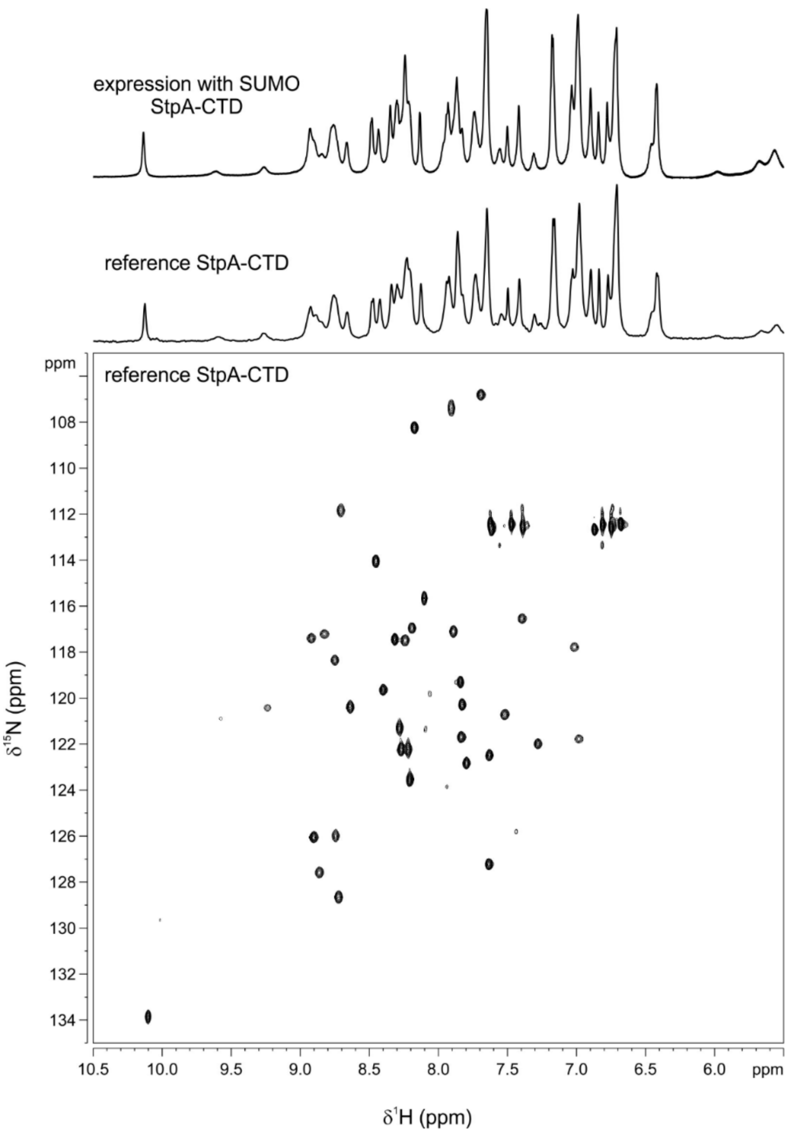

Figure S 9: (top) Amide ^{1}H NMR spectra of StpA-CTD via expression with SUMOstar vector and of reference StpA-CTD at 25°C. (below) [^{1}H,^{15}N]-HSQC spectra of reference StpA-CTD for the purpose of better resolution of the backbone and side-chain H^N resonances, confirming the folded state of StpA-CTD in both cases. Spectra recorded at 800 MHz/ 600 MHz Bruker NMR spectrometers. Concentrations c(expression with SUMO StpA-CTD)= 860 µM, c(reference StpA)=1000 µM.

4 RNA Sequences, Abbreviations, sample concentration

Table S 4: RNA abbreviations, sequences and modifications

RNA	Sequence	Modification
20mer	5'- GAC CGG AAG GUC CGC CUU CC -3'	
20mer caged	5'- GAC CG**G** AAG GUC CGC CUU CC -3'	(S)-NPE O6-nitrophenyl-ethyl
G6m1G	5'- GAC CG**G** AAG GUC CGC CUU CC -3'	m1G N1-methylguanosine
A2-DMA	5'- G**A**C CGG AAG GUC CGC CUU CC -3'	DMA N6-dimethyladenosin
5'-HP	5'- GAC CGG AAG GUC C -3'	
3'-HP	5'- CGG AAG GUC CGC CUU CC -3'	
5'-SS-OV	5'- GC CUU CC -3'	

5 NMR spectroscopy

NMR experiments were performed on Bruker NMR spectrometers with different probe heads listed in Table S 4. NMR experiments were performed with standard Bruker pulse sequences and spectra were recorded and analyzed with *TopSpin 3.5pl5-7*. All samples containing 10% D_2O and the same buffer: 50 mM BisTris, 25 mM NaCl, pH 6.4. 1D ^1H imino proton spectra were recorded using a jump return echo pulse sequence. Thermal equilibration for all samples at each temperature was done for at least 20 minutes before the experiments were recorded.

Table S 5. Spectrometers

Spectrometer	Probe Head
800 MHz	5 mm TCI cryo ^1H,^{15}N,^{13}C Z-GRD<br>5 mm TXO cryo ^{13}C,^1H,^{15}N, Z-GRD
700 MHz	5 mm QCI cryo ^1H,^{15}N,^{13}C,^{31}P Z-GRD
599 MHz	5 mm TCI cryo ^1H,^{15}N,^{13}C Z-GRD
600 MHz	CryoProbe Prodigy 5 mm TCI ^1H/^{19}F,^{15}N,^{13}C Z-GRD

Table S 6: RNA sample concentrations for NMR

RNA	conc. RNA alone	conc. RNA in complex
20mer	444 µM	73.34 µM
20mer caged	100 µM	100 µM
G6m1G	689 µM	200 µM
A2-DMA	800 µM	
5’-HP	483 µM	
3’-HP	1068 µM	

6 Data collection and analysis of thermodynamic parameters

6.1 Titration 20 nt bistable

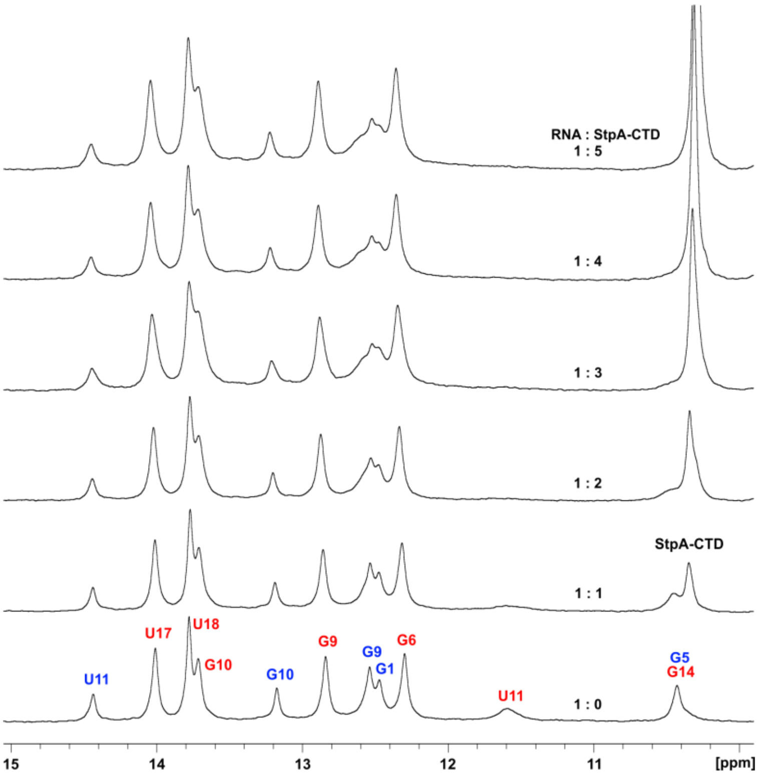

Figure S 10. ^{1}H-NMR Imino proton region of the 20 nt RNA (Figure 1e in the text) titrated with StpA-CTD until a ratio of 1:5 at 25°C. Buffer conditions: 50 mM BisTris, 25 mM NaCl, pH 6.4. Color-coded assignment of the resonances (5'-fold blue, 3'-fold red). Spectrometer: 599 MHz Bruker, c_{RNA}=100 µM – 73.34 µM, 4k ns.

Table S 7. Experimental values for different complex ratios of RNA and StpA-CTD. Obtained by division of the integral of imino resonances U17 from the 3'-fold and U11 from the 5'-fold.

RNA:StpA-CTD	K = int(U17)/int(U11)
1:0	2.94 ± 0,07
1:1	3.39 ± 0,11
1:2	4.02 ± 0,11
1:3	4.28 ± 0,14
1:4	4.28 ± 0,18
1:5	4.23 ± 0,12

6.2 Temperature Rows and free energy analysis

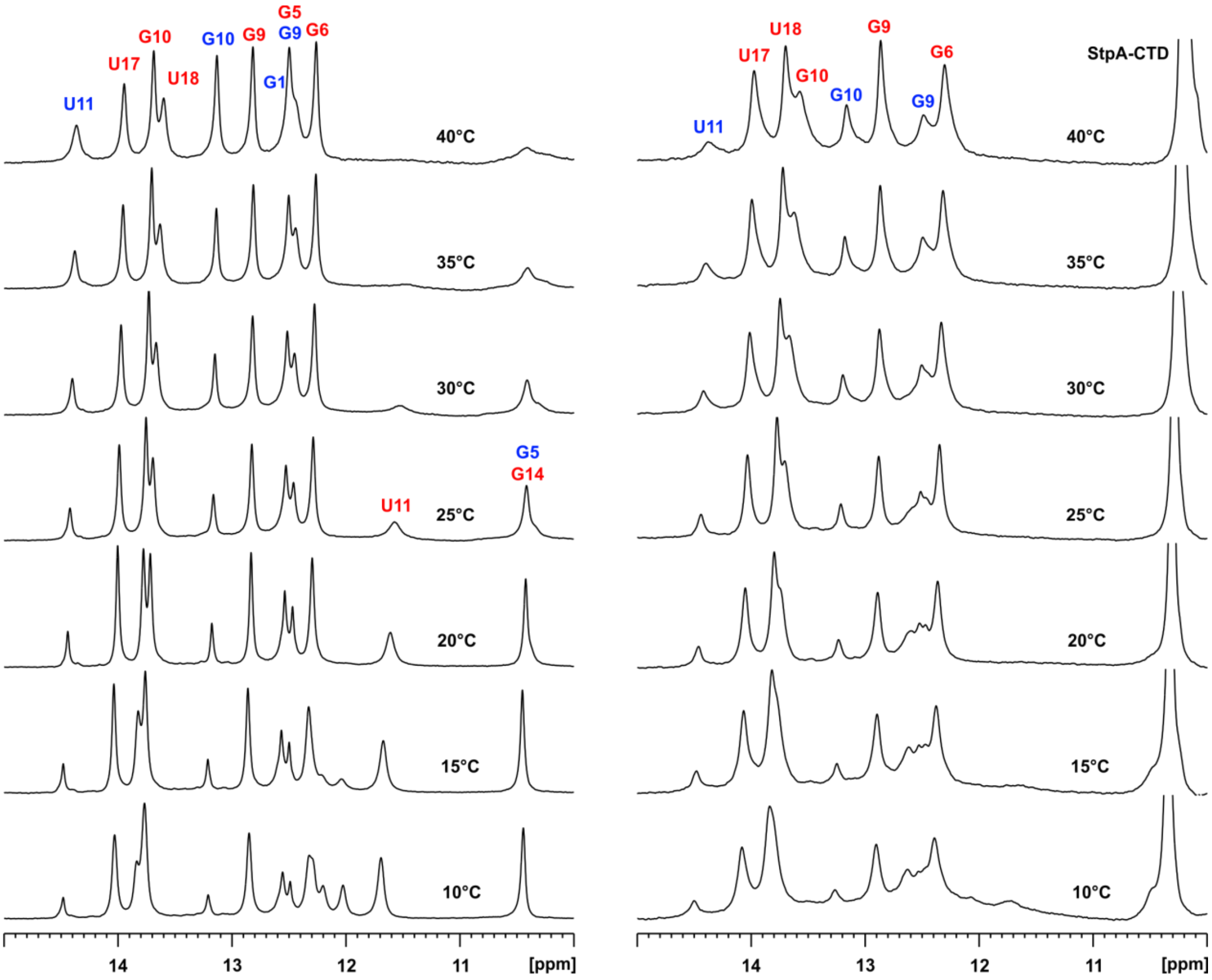

Figure S 11. ^{1}H-NMR Imino proton region of the 20 nt RNA (left) and the RNA in complex with 5 eq. of StpA-CTD (right). Buffer conditions: 50 mM BisTris, 25 mM NaCl, pH 6.4. Color-coded assignment of the resonances (5'-fold blue, 3'-fold red). Spectrometer: 900/ 599 MHz Bruker. c_{RNA}=477 µM/ 73.34 µM, 1k/ 6k.ns.

Table S 8. Experimental values of equilibrium constant K for the RNA and the RNA in complex with 5 eq. StpA-CTD at different temperatures. Obtained by division of the integral of imino resonances U17 from the 3'-fold and U11 from the 5'-fold. Also ΔG according to ΔG=-RTln(K) for RNA and complex at different temperatures.

	**K = Int(U17(3'-fold))/Int(U11(5'-fold))**		**ΔG [kJ/mol]**	
**Temperature [°C]**	**RNA**	**Complex 1:5**	**RNA**	**Complex 1:5**
**40**	1.46 ± 0.04	3.9 ± 0.4	-0.99 ± 0.08	-3.5 ± 0.3
**35**	1.90 ± 0.04	4.00 ± 0.17	-1.64 ± 0.05	-3.55 ± 0.12
**30**	2.48 ± 0.04	4.04 ± 0.19	-2.29 ± 0.04	-3.52 ± 0.12
**25**	3.09 ± 0.04	4.23 ± 0.15	-2.80 ± 0.03	-3.57 ± 0.09
**20**	3.60 ± 0.05	4.7 ± 0.2	-3.12 ± 0.03	-3.77 ± 0.11
**15**	4.37 ± 0.07	5.2 ± 0.4	-3.53 ± 0.04	-3.96 ± 0.15
**10**	5.22 ± 0.09	6.2 ± 0.4	-3.89 ± 0.04	-4.30 ± 0.16
**5**	6.64 ± 0.14	6.7 ± 0.4	-4.38 ± 0.05	-4.38 ± 0.13

7 ITC isothermal titration calorimetry

Table S 9: Overview if all ITC-experiments measured with StpA (c(StpA)=800/920 µM) and all RNA constructs (c(RNA=40µM)) at 5°C and 25°C in Buffer (50 mM BisTRIS, 25 mM NaCl, pH 6.4) and the derived binding parameters. Errors represent the standard deviation of 3 independent measurements.

RNA	T	K_D [µM]	N	ΔH [kJ/mol]	ΔS [J/mol/deg]	ΔG [kJ/mol]
**20mer**	5°C	9.4 ± 1.3	1.84 ± 0.02	-18.7 ± 1.9	29.2 ± 7.6	-26.8 ± 0.4
	25°C	12.31 ± 1.7	1.93 ± 0.05	-30.4 ± 0.6	-8.0 ± 3.0	-28.0 ± 0.4
**G6-m1G**	5°C	16.26 ± 0.7	1.93 ±0.07	-37.4 ± 0.4	-42.75 ± 1.75	-25.54 ± 0.08
	25°C	17.52 ± 1.3	1.80 ± 0.11	-43.1 ± 0.8	-53.6 ± 1.9	-27.1 ± 0.3
**A2-DMA**	5°C	8.31 ± 0.8	2.21 ± 0.26	-17.10 ± 0.12	35.9 ± 0.7	-27.1 ± 0.3
	25°C	13.6 ± 1.6	2.33 ± 0.17	-24.3 ± 0.8	12.1 ± 2.6	-27.9 ± 0.3
**5'-HP**	5°C	13.3 ± 3.7	1.74 ± 0.06	-29.6 ± 0.5	-12.5 ± 3.1	-25.8 ± 0.7
	25°C	24.7 ± 0.6	1.69 ± 0.05	-36.6 ± 0.8	-34.5 ± 2.4	-26.30 ± 0.06
**3'-HP**	5°C	11.3 ± 1.4	1.78 ± 0.18	-18.6 ± 0.7	28.0 ± 2.0	-26.4 ± 0.3
	25°C	16.15 ± 2.6	1.74 ± 0.05	-28.2 ± 0.3	-2.7 ± 1.2	-27.4 ± 0.5

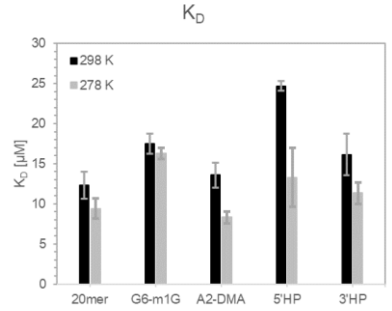

Figure S 12: Binding affinity expressed as K_D and the free energy of the binding process of StpA to all RNA constructs at 5°C and 25°C. Error bars represent the standard deviation of 3 independent measurements

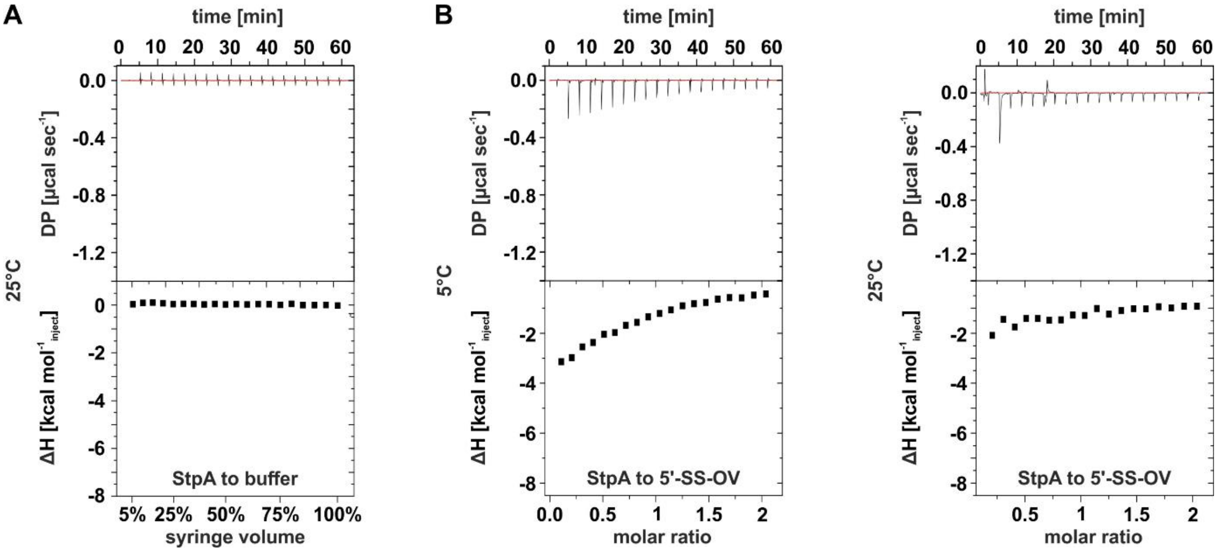

Figure S 13. (A) ITC thermogram for StpA-CTD (920 µM) titrated to buffer (50 mM BisTRIS, 25 mM NaCl, pH 6.4) at 25°C, resulting in a baseline. (B) ITC thermograms for StpA-CTD (400 µM) titrated to 5'-SS-OV (40 µM) at 5°C and 25°C.

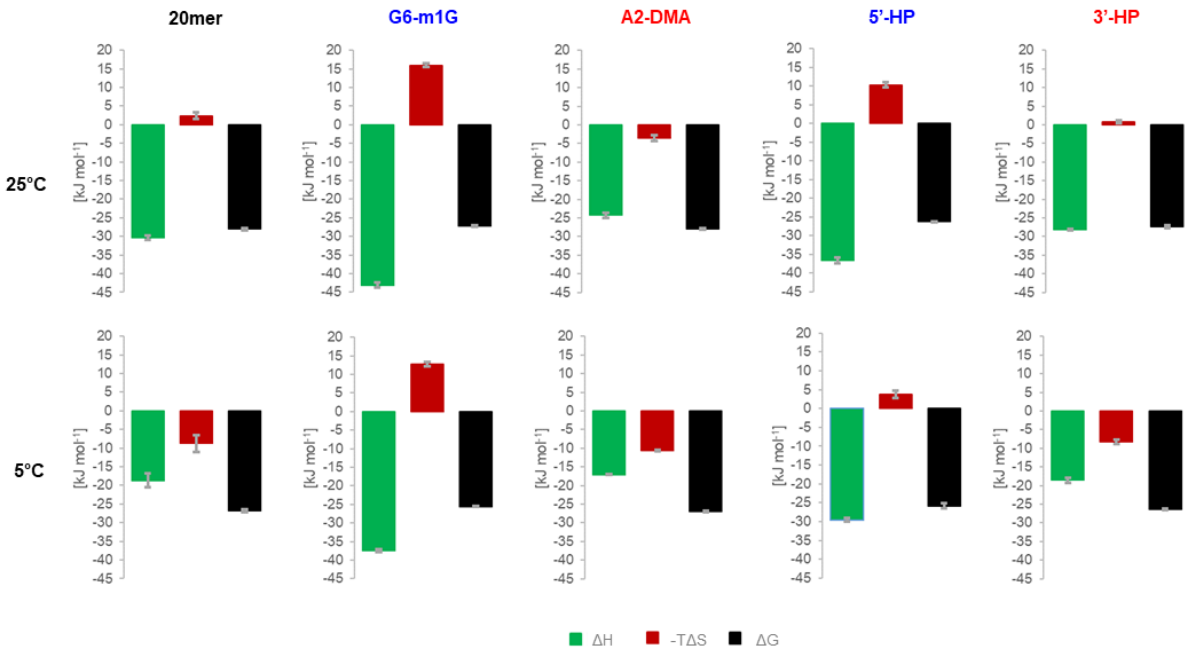

Figure S 14: Thermodynamic signatures of StpA binding to different RNA constructs at 5°C and 25°C. ΔH is represented by green bars, -TΔT by red bars and ΔG by black bars. Error bars represent the standard deviation of 3 independent measurements.

8 Data collection and analysis real-time NMR

8.1 Degree of photolysis

Table S 10. Degree of photolysis p [%] after the first laser-pulse was determined from the appearing signals, by comparing the signals after the first laser pulse of 355 nm, 1 s and 4 W with signals of the unmodified RNA or complex at the same temperature. The RNA concentration was 100 µM.

**Int(U11(5'))/Int(U17(3')) - Degree of Photolysis p [%]**										
	**RNA**					**complex**				
**Temperature [°C]**	**K (1s)**	**% 5' (1s)**	**K (unmodified)**	**% 5' (unmodified)**	**% p**	**K (1s)**	**% 5' (1s)**	**K (unmodified)**	**% 5' (unmodified)**	**% p**
**5**	3.4	22.8	6.9	12.7	**89.9**	2.6	27.9	6.5	13.3	**85.4**
**10**	1.0	49.1	5.8	14.6	**65.5**	1.9	34.3	6.0	14.4	**80.0**
**17**	1.2	45.7	4.3	18.8	**73.0**	0.7	59.9	5.1	16.4	**56.5**
**25**	0.5	65.3	3.4	22.7	**57.4**	1.2	46.4	4.5	18.1	**71.7**

8.2 Analysis of uncaged RNA sample

An analytical denaturing 15% PAGE (polyacrylamide) was performed to ensure that laser light, StpA-CTD or PEG-8000 do not degrade uncaged RNA. Uncaged RNAs do not show degradation, also in complex with StpA-CTD or in presence of PEG-8000. After uncaging the RNA is at the same horizontal position as unmodified RNA.

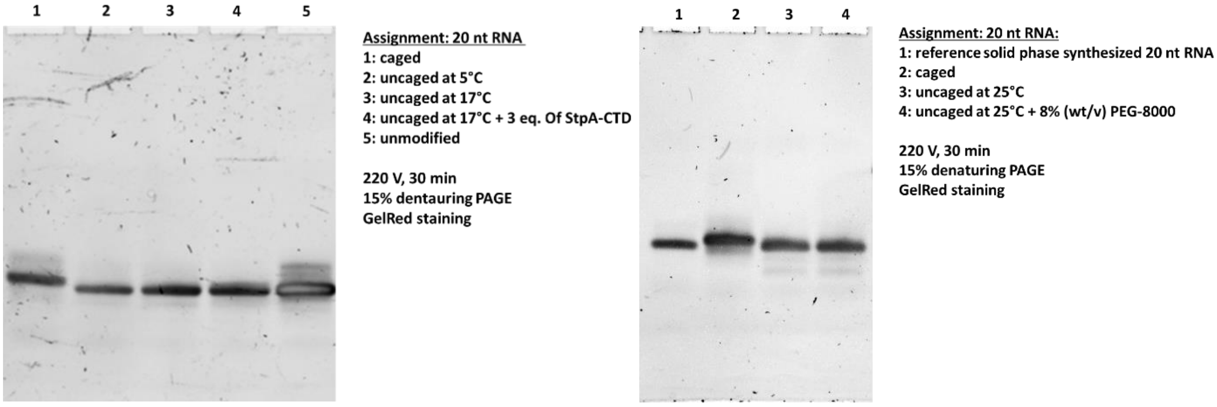

Figure S 15. Quality control of uncaged RNA, uncaged RNA in complex with StpA and in presence of PEG-8000 at different temperatures by analytical denaturing 15% PAGE.

8.3 Fit of kinetics rates

The individual kinetic traces of the imino proton signals were extracted from the pseudo 2D kinetic spectra. Fitting of the traces was done according to Wenter *et al*. (1) dependent on the corresponding equilibrium constant K (see SI table S8) for a reversible unimolecular reaction:

normalized signal$(3')_t$ = $K/(K+1)*(1 - e^{(-k(5'\text{-}3')t*(1+1/K))})$

normalized signal$(5')_t$ = $1/(K+1)*(1 + K\ e^{(-k(5'\text{-}3')t*(1+1/K))})$

$k_{3'\text{-}5'} = k_{5'\text{-}3'}/K$

Table S 11. Refolding kinetics data for the **RNA** at different temperatures. Rate constants $k_{5'\text{-}3'}$ and calculated from these rate constants $k_{3'\text{-}5'}$ according to K= $k_{5'\text{-}3'}/k_{3'\text{-}5'}$, both given with fit error. Error of mean values is the standard deviation of all corresponding rates

**RNA**	**U11**	**U17**	**G9**	**G10**	**mean values all bases**
**Temperature [°C]**	**$k_{5'\text{-}3'}$ [10^{-3} s^{-1}]**	**$k_{5'\text{-}3'}$ [10^{-3} s^{-1}]**	**$k_{5'\text{-}3'}$ [10^{-3} s^{-1}]**	**$k_{5'\text{-}3'}$ [10^{-3} s^{-1}]**	**$k_{5'\text{-}3'}$ [10^{-3} s^{-1}]**
**5**	10.3±0.3	13.8±0.6	11.0±0.5	9.8±0.4	11±3
**10**	17.1±1.2	14.6±1.1	14±2	23.9.6±2	16±6
**17**	41±2	52±5	56±7	58±7	55±8
**25**	300±5	280±50	200±60	280±50	240±40
**Temperature [°C]**	**$k_{3'\text{-}5'}$ [10^{-3} s^{-1}]**	**$k_{3'\text{-}5'}$ [10^{-3} s^{-1}]**	**$k_{3'\text{-}5'}$ [10^{-3} s^{-1}]**	**$k_{3'\text{-}5'}$ [10^{-3} s^{-1}]**	**$k_{3'\text{-}5'}$ [10^{-3} s^{-1}]**
**5**	1.55±0.05	2.08±0.10	1.48±0.07	1.66±0.08	1.7±0.3
**10**	3.3±0.3	2.8±0.3	2.6±0.5	4.6±0.6	3.1±1.2
**17**	10.3±0.6	13.1±1.2	14.1±1.7	14.6±1.8	14±2
**25**	96±19	91±19	64±9	90±20	84±15

Table S 12. Refolding kinetics data for the RNA in **complex** with three eq. of StpA-CTD at different temperatures. Rate constants $k_{5'-3'}$ and calculated from these rate constants $k_{3'-5'}$ according to K= $k_{5'-3'}/k_{3'-5'}$, both given with fit error. Error of mean values is the standard deviation of all corresponding rates

complex	U11	U17	G9	G10	mean values all bases
Temperature [°C]	$k_{5'-3'}$ [10^{-3} s^{-1}]	$k_{5'-3'}$ [10^{-3} s^{-1}]	$k_{5'-3'}$ [10^{-3} s^{-1}]	$k_{5'-3'}$ [10^{-3} s^{-1}]	$k_{5'-3'}$ [10^{-3} s^{-1}]
**5**	27.7±3	20.4±1.8	24.2±3	24.9±2	25±3
**10**	34.4±3	23.3±2	29.6±4	37.6±4	30.4±5
**17**	86±13	140±40	180±170	90±30	100±30
**25**	180±30	200±40	220±130	150±50	190±40
**Temperature [°C]**	$k_{3'-5'}$ [10^{-3} s^{-1}]	$k_{3'-5'}$ [10^{-3} s^{-1}]	$k_{3'-5'}$ [10^{-3} s^{-1}]	$k_{3'-5'}$ [10^{-3} s^{-1}]	$k_{3'-5'}$ [10^{-3} s^{-1}]
**5**	4.2±0.3	3.1±0.3	3.6±0.5	3.7±0.3	3.7±0.6
**10**	5.5±0.5	3.8±0.3	4.8±0.7	6.1±0.7	4.9±0.9
**17**	17±2	29±9	40±30	19±7	21±7
**25**	42±7	48±11	50±.30	56±13	46±10

8.4 Kinetics under molecular Crowding conditions with PEG-8000

For these experiments at 278 K and 298 K RNA and RNA with 8 %(w/v) PEG-800 was uncaged as described previous. The kinetic traces were evaluated as described in SI 7.3. The kinetic traces for PEG-8000 and for the RNA alone, both were fitted with the corresponding equlibirum constant for the RNA ate the corresponding temperature (see SI Table S 8)

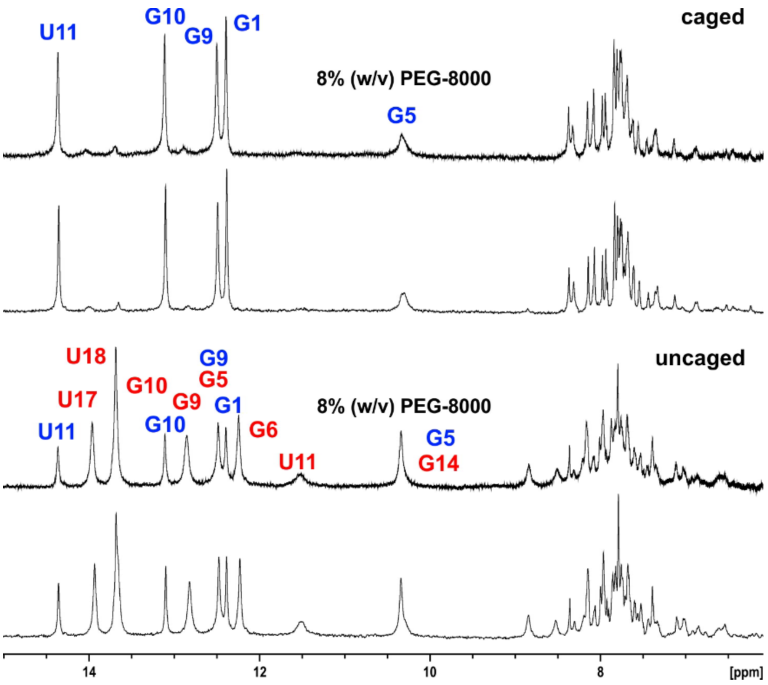

Figure S 16. ^{1}H NMR spectra of imino and aromatic protons of the 20 nt RNA alone (light lines) and with 8% (w/v) PEG-800 (bold lines) (c(RNA)= 100 µM). Caged: O^6-(S)-NPE modified guanosine at position G6, uncaged: same samples after photolysis by a laser pulse (λ=355 nm, P=4 W, t=1 s) after 30 min of equilibration at 25°C.

Table S 13. Refolding kinetics data for the RNA in presence of 8% (w/v) PEG-8000at different temperatures. Rate constants $k_{5'-3'}$ and calculated from these rate constants $k_{3'-5'}$ according to K= $k_{5'-3'}/k_{3'-5'}$, both given with fit error. Error of mean values is the standard deviation of all corresponding rates.

**PEG-8000**	**U11**	**U17**	**G9**	**G10**	**mean values all bases**
**Temperature [°C]**	**$k_{5'-3'}$ [10^{-3} s^{-1}]**	**$k_{5'-3'}$ [10^{-3} s^{-1}]**	**$k_{5'-3'}$ [10^{-3} s^{-1}]**	**$k_{5'-3'}$ [10^{-3} s^{-1}]**	**$k_{5'-3'}$ [10^{-3} s^{-1}]**
**5**	16.5±0.5	14.6±0.6	10.4±0.4	23.4±0.4	16.4±4.6
**25**	350±36	177±14	271±47	397±57	261±93
**Temperature [°C]**	**$k_{3'-5'}$ [10^{-3} s^{-1}]**	**$k_{3'-5'}$ [10^{-3} s^{-1}]**	**$k_{3'-5'}$ [10^{-3} s^{-1}]**	**$k_{3'-5'}$ [10^{-3} s^{-1}]**	**$k_{3'-5'}$ [10^{-3} s^{-1}]**
**5**	2.48±0.09	2.20±0.10	1.57±0.10	3.52±0.06	2.5±0.7
**25**	113±12	58±5	88±16	129±19	67±44

Table S 14. Refolding kinetics data for the RNA at 5°C and 25°C. Rate constants $k_{5'-3'}$ and calculated from these rate constants $k_{3'-5'}$ according to K= $k_{5'-3'}/k_{3'-5'}$, both given with fit error. Error of mean values is the standard deviation of all corresponding rates.

**RNA**	**U11**	**U17**	**G9**	**G10**	**mean values all bases**
**Temperature [°C]**	**$k_{5'-3'}$ [10^{-3} s^{-1}]**	**$k_{5'-3'}$ [10^{-3} s^{-1}]**	**$k_{5'-3'}$ [10^{-3} s^{-1}]**	**$k_{5'-3'}$ [10^{-3} s^{-1}]**	**$k_{5'-3'}$ [10^{-3} s^{-1}]**
**5**	10.2±0.2	13.4±0.4	9.3±0.6	13.4±0.04	11.8±3.2
**25**	284±26	203±17	224±36	281±34	239±55
**Temperature [°C]**	**$k_{3'-5'}$ [10^{-3} s^{-1}]**	**$k_{3'-5'}$ [10^{-3} s^{-1}]**	**$k_{3'-5'}$ [10^{-3} s^{-1}]**	**$k_{3'-5'}$ [10^{-3} s^{-1}]**	**$k_{3'-5'}$ [10^{-3} s^{-1}]**
**5**	1.54±0.03	2.02±0.06	1.40±0.10	2.02±0.06	1.8±0.5
**25**	92±9	66±6	72±12	91±11	70±30

Table S 15. Acceleration factor of refolding of 20 nt bistable RNA in presence of 8% (w/v) PEG-8000 or 3 equivalents StpA for rate constants $k_{5'-3'}$ and $k_{3'-5'}$. Error of the acceleration factor was calculated by propagation of the percentage error of the individual rates.

acceleration factor	PEG-8000		StpA	
	5°C	25°C	5°C	25°C
$k_{5'-3'}$	1.39±0.39	1.07±0.37	2.17±0.43	0.8±0.10
$k_{3'-5'}$	1.39±0.38	0.98±0.46	2.21±0.44	0.55±0.04

9 Data collection and analysis of base pairs stabilities by NMR

9.1 ^{1}H 1D characterization supplementary RNA constructs

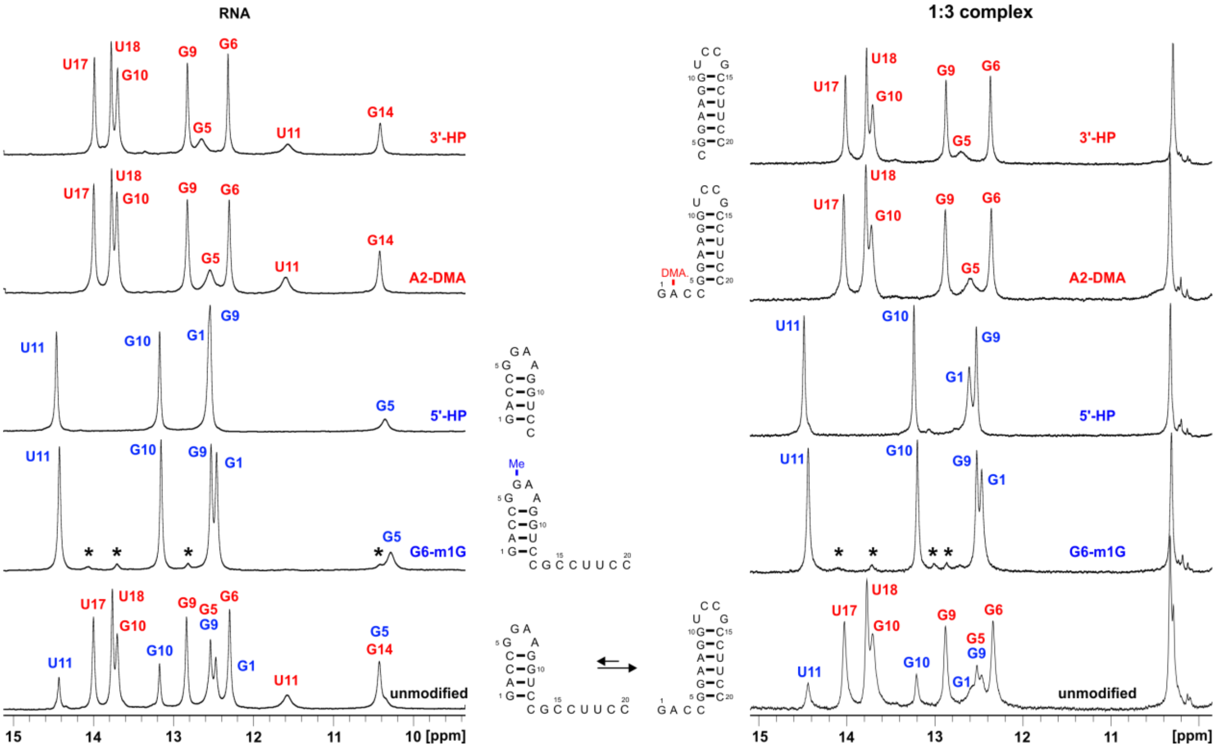

Figure S 17: ^{1}H-NMR imino spectra of the different RNA constructs (left) and the different RNA constructs in complex with three eq. of StpA-CTD (right). Recorded at 600/ 800 MHz with a jump-return echo sequence for water suppression at 25°C. In the middle, the RNA structures are shown with corresponding spectra aside. Bottom: unmodified RNA with imino signals from both conformations color-coded. G6-m1G and 5'-HP with signals just from the 5'-fold and A2-DMA and 3'-HP with signals from the 3'-fold. All constructs in 50 mM BisTris, 25 mM NaCl, pH 6.4. $c_{unmodified}$=444 µM (1k ns), $c_{G6\text{-}m1G}$=689 µM (256 ns), $c_{5'\text{-}HP}$=483 µM (1k ns), $c_{A2\text{-}DMA}$=800 µM (1k ns), $c_{3'\text{-}HP}$=1068 µM (64 ns), $c_{all\ complexes}$=200 µM (1k ns). * 5% of 3'-fold present.

9.2 Water exchange rates

Pseudo 2D water exchange NMR experiments were conducted and evaluated as described in Rinnenthal et al. (3).

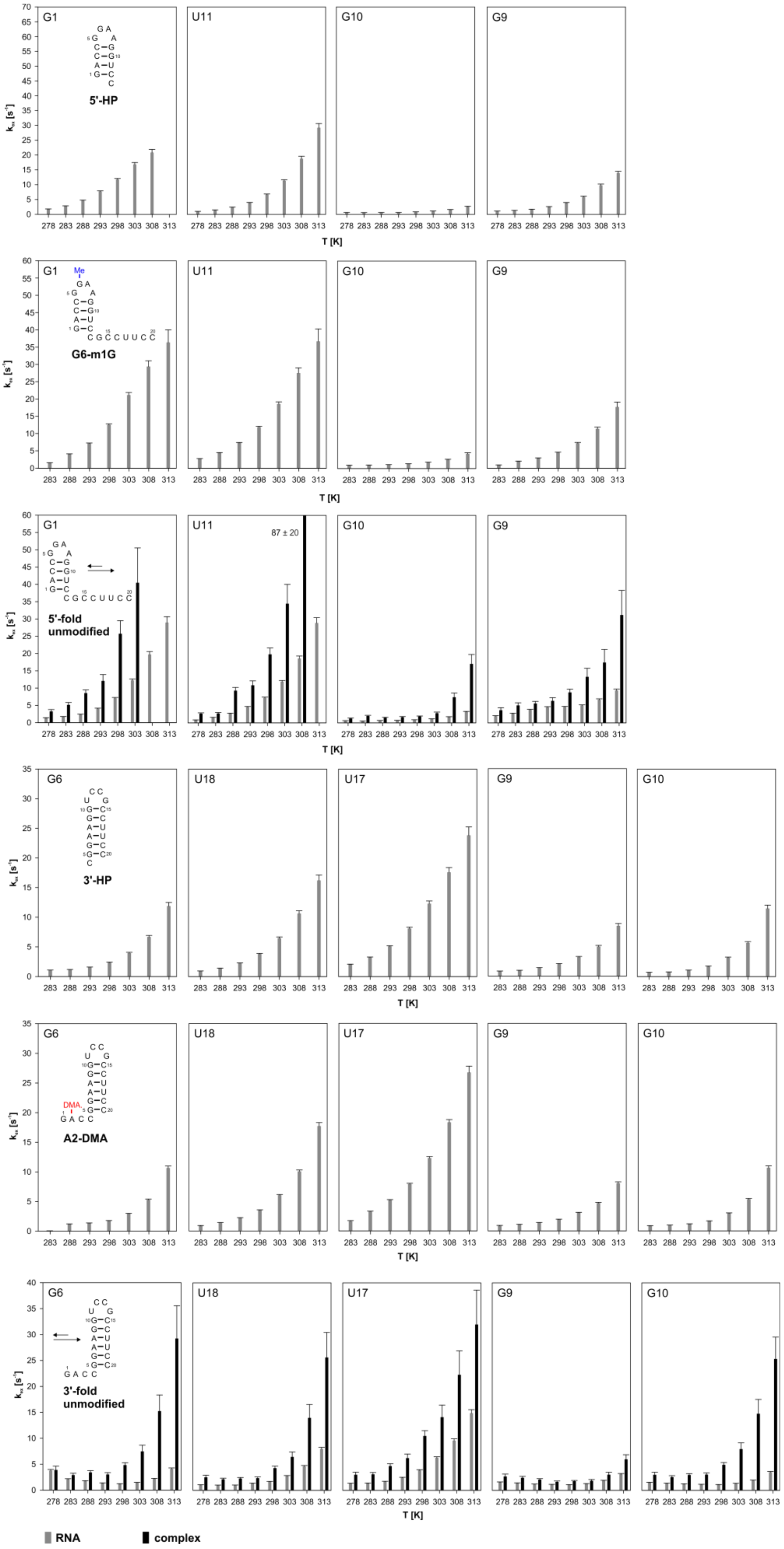

G1
U11
G10
G9
5'-HP
G6-m1G
Me
5'-fold unmodified
87 ± 20
G6
U18
U17
3'-HP
A2-DMA
DMA
3'-fold unmodified
k_{ex} [s^{-1}]
T [K]
RNA
complex

Figure S 18. Temperature dependence of the imino proton exchange rate k_{ex} for the individual imino protons of the different RNA systems based on the 3'-fold and 5'-fold. Color code: gray data points for the RNA and black for the RNA in complex with 3 eq. of StpA-CTD. Error bars represent the errors of the fit.

Table S 16. ΔH_{diss}, ΔS_{diss} and ΔG_{diss} (T=25°C) for the base pair opening of individual nucleobases within the truncated **3'-HP RNA** alone and in complex with 3 eq. of StpA-CTD. Errors represent the standard deviation, calculated with experimental errors of k_{ex}.

3'-HP	ΔH_{diss} [kJ/mol]	ΔS_{diss} [J/mol*K]	ΔG_{diss} [kJ/mol] [T=298K]
**U17**	18.76±1.59	14.20±5.63	14,53±0.16
**U18**	41.33±1.35	83.48±4.77	16.46±0.08
**G10**	78.99±1.07	200.26±3.84	19.31±0.07
**G9**	51.61±0.74	110.15±2.77	18.79±0.09
**G6**	57.28±1.25	131.21±4.42	18.18±0.07

Table S 17. ΔH_{diss}, ΔS_{diss} and ΔG_{diss} (T=25°C) for the base pair opening of individual nucleobases within the trapped **A2-DMA** RNA alone and in complex with 3 eq. of StpA-CTD. Errors represent the standard deviation, calculated with experimental errors of k_{ex}.

A2-DMA	ΔH_{diss} [kJ/mol]	ΔS_{diss} [J/mol*K]	ΔG_{diss} [kJ/mol] [T=298K]
**U17**	26.59±1.10	40.18±3.51	14.62±0.05
**U18**	52.45±1.10	119.17±3.48	16.93±0.05
**G10**	76.25±0.071	190.83±2.55	19.38±0.05
**G9**	51.20±0.41	108.36±1.56	18.91±0.07
**G6**	74.08±0.89	183.89±3.10	19.28±0.05

Table S 18. ΔH_{diss}, ΔS_{diss} and ΔG_{diss} (T=25°C) for the base pair opening of individual nucleobases within the **unmodified 3'-fold RNA** alone and in complex with 3 eq. of StpA-CTD. Errors represent the standard deviation, calculated with experimental errors of k_{ex}.

3'-fold	ΔH_{diss} [kJ/mol]	ΔS_{diss} [J/mol*K]	ΔG_{diss} [kJ/mol] [T=298K]
**RNA**			
**U17**	36.83±0.45	68.28±1.82	16.49±0.10
**U18**	58.41±0.11	131.73±0.71	19.15±0.11
**G10**	56.82±2.11	118.41±6.39	21.53±0.28
**G9**	46.10±2.36	83.05±7.19	21.35±0.29
**G6**	70.57±0.32	164.11±0.65	21.67±0.13
**complex**			
**U17**	22.52±3.16	29.04±11.90	13.87±0.27
**U18**	62.44±2.41	154.65±9.34	16.35±0.36
**G10**	47.86±0.51	107.54±3.11	15.82±0.39
**G9**	27.48±3.86	22.92±11.07	18.86±0.60
**G6**	66.50±3.92	168.33±14.41	16.33±0.36

Table S 19. ΔH_{diss}, ΔS_{diss} and ΔG_{diss} (T=25°C) for the base pair opening of individual nucleobases within the truncated 5'-HP RNA alone and in complex with 3 eq. of StpA-CTD. Errors represent the standard deviation, calculated with experimental errors of k_{ex}.

5'-HP	ΔH_{diss} [kJ/mol]	ΔS_{diss} [J/mol*K]	ΔG_{diss} [kJ/mol] [T=298K]
**U11**	39.44±0.94	82.43±3.42	14.88±0.09
**G10**	85.21±3.60	206.79±11.11	23.59±0.29
**G1**	16.22±1.04	8.27±3.78	13.76±0.09
**G9**	33.59±0.31	57.08±1.36	16.58±0.10

Table S 20. ΔH_{diss}, ΔS_{diss} and ΔG_{diss} (T=25°C) for the base pair opening of individual nucleobases within the trapped G6-m1G RNA alone and in complex with 3 eq. of StpA-CTD. Errors represent the standard deviation, calculated with experimental errors of k_{ex}.

G6-m1G	ΔH_{diss} [kJ/mol]	ΔS_{diss} [J/mol*K]	ΔG_{diss} [kJ/mol] [T=298K]
**U11**	20.84±3.31	25.07±11.32	13.37±0.07
**G10**	49.80±2.21	98.13±7.64	20.56±0.08
**G9**	36.75±3.06	69.10±10.44	16.16±0.05
**G1**	32.43±1.85	63.65±6.46	13.46±0.08

Table S 21. ΔH_{diss}, ΔS_{diss} and ΔG_{diss} (T=25°C) for the base pair opening of individual nucleobases within the unmodified 5'-fold RNA alone and in complex with 3 eq. of StpA-CTD. Errors represent the standard deviation, calculated with experimental errors of k_{ex}.

5'-fold	ΔH_{diss} [kJ/mol]	ΔS_{diss} [J/mol*K]	ΔG_{diss} [kJ/mol] [T=298K]
**RNA**			
**U11**	35.30±1.12	69.06±4.01	14.72±0.09
**G10**	93.23±1.59	234.23±4.73	23.43±0.20
**G9**	**16.69**±0.11	**0.0**±0.0	**16.69**±0.12
**G1**	35.34±1.18	68.89±4.26	14.81±0.10
**complex**			
**U11**	80.52±12.17	228.98±41.60	12.28±0.23
**G10**	101.49±9.54	275.54±29.18	19.38±0.45
**G9**	22.80±4.09	28.84±15.07	14.21±0.45
**G1**	41.78±7.14	100.98±16.34	11.69±0.56

9.3 Temperature dependence of destabilization induced by StpA-CTD

Table S 22. $\Delta\Delta G_{Diss}$ difference in base pair stabilities within the RNA alone and the RNA in complex with 3 eq. of StpA-CTD at different temperatures between 5°C and 40°C.

	$\Delta\Delta G_{Diss}$ [%, complex/RNA]										
	**5'-fold**					**3'-fold**					
**T [°C]**	**G1**	**U11**	**G10**	**G9**	**mean value**	**G6**	**U18**	**U17**	**G9**	**G10**	**mean value**
**5**	84.7	104.7	88.5	88.6	**76.3**	89.3	80.9	94.6	75.2	84.7	**104.7**
**10**	83.3	99.7	87.3	87.7	**75.8**	88.4	81.7	94.1	74.8	83.3	**99.7**
**15**	81.9	94.5	85.9	86.9	**75.3**	87.5	82.5	93.6	74.4	81.9	**94.5**
**20**	80.5	89.1	84.4	86.0	**74.8**	86.5	83.3	93.1	73.9	80.5	**89.1**
**25**	78.9	83.4	82.7	85.1	**74.2**	85.4	84.1	92.5	73.5	78.9	**83.4**
**30**	77.3	77.5	80.9	84.3	**73.5**	84.3	85.0	92.0	73.0	77.3	**77.5**
**35**	75.6	71.2	78.8	83.4	**72.8**	83.0	85.9	91.4	72.4	75.6	**71.2**
**40**	73.8	64.6	76.6	82.5	**72.0**	81.7	86.9	90.8	71.9	73.8	**64.6**

References

1. Wenter,P., Fürtig,B., Hainard,A., Schwalbe,H. and Pitsch,S. (2005) Kinetics of photoinduced RNA refolding by real-time NMR spectroscopy. *Angew. Chemie - Int. Ed.*, **44**, 2600–2603.
2. Helmling,C., Klötzner,D.-P., Sochor,F., Mooney,R.A., Wacker,A., Landick,R., Fürtig,B., Heckel,A. and Schwalbe,H. (2018) Life times of metastable states guide regulatory signaling in transcriptional riboswitches. *Nat. Commun.*, **9**, 944.

3. Rinnenthal,J., Klinkert,B., Narberhaus,F. and Schwalbe,H. (2010) Direct observation of the temperature-induced melting process of the Salmonella fourU RNA thermometer at base-pair resolution. *Nucleic Acids Res.*, **38**, 3834–3847.
